# Capacity of non-invasive hepatic fibrosis algorithms to replace transient elastography to exclude cirrhosis in people with hepatitis C virus infection: A multi-centre observational study

**DOI:** 10.1371/journal.pone.0192763

**Published:** 2018-02-13

**Authors:** Melissa Louise Kelly, Stephen M. Riordan, Rohan Bopage, Andrew R. Lloyd, Jeffrey John Post

**Affiliations:** 1 Department of Infectious Diseases, Prince of Wales Hospital, Randwick, NSW, Australia; 2 Department of Medicine, The Albion Centre, Surry Hills, NSW, Australia; 3 Population Heath, Justice Health & Forensic Mental Health Network, Malabar, NSW Australia; 4 Prince of Wales Clinical School, School of Medicine, UNSW, Kensington, NSW, Australia; 5 Gastrointestinal and Liver Unit, Prince of Wales Hospital, Randwick, NSW, Australia; 6 The Kirby Institute, Viral Immunology Systems Program, Kensington, NSW, Australia; Harvard Medical School, UNITED STATES

## Abstract

**Introduction:**

Achievement of the 2030 World Health Organisation (WHO) global hepatitis C virus (HCV) elimination targets will be underpinned by scale-up of testing and use of direct-acting antiviral treatments. In Australia, despite publically-funded testing and treatment, less than 15% of patients were treated in the first year of treatment access, highlighting the need for greater efficiency of health service delivery. To this end, non-invasive fibrosis algorithms were examined to reduce reliance on transient elastography (TE) which is currently utilised for the assessment of cirrhosis in most Australian clinical settings.

**Materials and methods:**

This retrospective and prospective study, with derivation and validation cohorts, examined consecutive patients in a tertiary referral centre, a sexual health clinic, and a prison-based hepatitis program. The negative predictive value (NPV) of seven non-invasive algorithms were measured using published and newly derived cut-offs. The number of TEs avoided for each algorithm, or combination of algorithms, was determined.

**Results:**

The 850 patients included 780 (92%) with HCV mono-infection, and 70 (8%) co-infected with HIV or hepatitis B. The mono-infected cohort included 612 men (79%), with an overall prevalence of cirrhosis of 16% (125/780). An ‘APRI’ algorithm cut-off of 1.0 had a 94% NPV (95%CI: 91–96%). Newly derived cut-offs of ‘APRI’ (0.49), ‘FIB-4’ (0.93) and ‘GUCI’ (0.5) algorithms each had NPVs of 99% (95%CI: 97–100%), allowing avoidance of TE in 40% (315/780), 40% (310/780) and 40% (298/749) respectively. When used in combination, NPV was retained and TE avoidance reached 54% (405/749), regardless of gender or co-infection.

**Conclusions:**

Non-invasive algorithms can reliably exclude cirrhosis in many patients, allowing improved efficiency of HCV assessment services in Australia and worldwide.

## Introduction

The World Health Organisation (WHO) has called for the elimination of hepatitis C virus (HCV) infection by 2030 [1]. Improvements in the efficiency of patient assessment and treatment will be essential to achieve this aim. In Australia there are approximately 230,000 people living with chronic HCV infection [2], with over 10,000 new diagnoses made in 2015 [3]. The major risk factor for HCV acquisition in Australia is receptive syringe sharing amongst people who inject drugs [3], with over 50% of those attending needle and syringe programs testing positive for HCV.

Less than 30,000 people with HCV infection have been treated since March 2016, despite effective, safe and convenient HCV direct acting antivirals (DAA) being available to all HCV-infected Australians, including prisoners, at low or no cost [3–5]. To date, HCV treatment has been undertaken predominantly in tertiary settings. Care will need to be shifted predominantly to primary care to rapidly scale-up treatment which will reduce the complications of cirrhosis and public health expense, as well as allow the achievement of the WHO elimination targets [1, 6].

Assessment of the presence or absence of cirrhosis is important, as those with cirrhosis may require an altered treatment regimen, and require screening for complications including oesophageal varices and hepatocellular carcinoma (HCC) [4, 7–10]. Reliance on liver biopsy to determine the presence or absence of cirrhosis would severely mitigate against achieving WHO treatment targets, due to both limited availability of resources required for this procedure and poor patient acceptance. Consequently, in Australia, this assessment is most commonly undertaken by transient fibro-elastography (TE), which, due to its high diagnostic accuracy, has replaced the need for liver biopsy for fibrosis assessment in almost all patients [11–12]. TE uses transducer-induced vibration waves to define the stage of fibrosis [13]. However, it requires specifically trained clinicians and dedicated equipment that can limit the timely availability of this diagnostic modality [4, 13].

A number of non-invasive hepatic fibrosis scoring algorithms, based on standard laboratory measures and demographic data, have also been developed to assess hepatic fibrosis [14]. Most of these published algorithms use readily-available and relatively inexpensive parameters such as biochemical liver function tests, coagulation profile, platelet count and age. Some are proprietary tests with additional costs in addition to standard pathology testing, making their ‘real world’ utility uncertain [14].

Most of the algorithms were developed to positively identify cirrhosis, in order to avoid liver biopsy with published positive predictive values (PPV) ranging from 31–99% [13–25]. TE is superior to APRI for the prediction of cirrhosis with a diagnostic odds ratio of 66.5 vs. 7.5 [26]. Several studies have examined performance of the algorithms in exclusion of cirrhosis with published negative predictive values (NPV) between 38–100% [13, 14, 16, 23–25]. In addition, most studies have excluded patients co-infected with HIV or HBV, although a recent Vietnamese study showed good concordance between TE-defined fibrosis and the aspartate aminotransferase (AST) to platelet ratio index (APRI) and Fibrosis-4 (FIB-4) score in HCV/HIV co-infected patients [27].

This study aimed to examine the NPV of seven non-invasive algorithms (singly and in combination) to reliably and simply exclude cirrhosis, and determine the proportion of TE that could be safely avoided in the assessment of patients with chronic HCV infection for DAA treatment.

## Materials and methods

A prospective and retrospective, multi-centre, observational cohort study was conducted with derivation and validation cohorts across three sites: a tertiary referral public hospital (Prince of Wales Hospital, New South Wales (NSW)), a public sexual health clinic (The Albion Centre, NSW) and the NSW state-wide prison hepatitis service run by the Justice Health and Forensic Mental Health Network (JH&FMN). The study population included consecutive patients with HCV infection who were assessed at the study sites for initiation of HCV therapy between December 2014 and December 2016.

The study protocol was approved by appropriate Human Research Ethics Committees (HREC) of all facilities (South Eastern Sydney Local Health District HREC and NSW JH&FMN HREC). Written consent was deemed unnecessary as the data were analysed in a de-identified format. The study was carried out in accordance with the Helsinki Declaration.

The data recorded included recruitment site, age, gender, co-infection status (HBV, HIV, HCV genotype, aspartate aminotransferase (AST) level and upper limit of normal (ULN), alanine aminotransferase level (ALT), ɣ-glutamyl transpeptidase (GGT) level, platelet count, international normalised ratio (INR), and total cholesterol level (fasting not required). Pathology testing was performed for each patient by the diagnostic laboratory linked to each facility. Clinical sites provided de-identified data to the research team for collation and analysis.

TE was undertaken with FibroScan® models 402 or 502. A ‘XL probe’ was utilised in patients who weighed more than 90 kilograms. The pressure reading on TE in kilopascals (kPa) was used to define the degree of hepatic fibrosis including cirrhosis. In keeping with Australian guidelines [4], in mono-infected patients the kPa cut-off for cirrhosis was >12.5kPa, while patients with HIV or HBV co-infection were considered to have cirrhosis with kPa values of >12.0kPa [13, 28–29]. TE was undertaken by practitioners trained in its use. Subjects were excluded if their measurement of fibrosis by TE had an interquartile range (IQR) from 10 measurements of 21% or more. Data were only recorded if the TE and the pathology results were undertaken prior to the commencement of HCV therapy and were within 4 months of each other, except for HCV genotype. When more than one set of pathology results were available, the results closest to the time of TE were recorded.

The non-invasive algorithms evaluated included: AST to Platelet Ratio Index (APRI) [15]; the cirrhosis discriminant score (CDS) proposed by Bonacini *et al* [17], Fibrosis-4 (FIB-4) [18], the Forns’ Index [19], the Göteborg University Cirrhosis Index (GUCI) [20], the King’s Score [21], and the Lok Index [22]. **Table 1** identifies the parameters each algorithm utilises.

**Table 1:** Indices utilised in the non-invasive algorithms included.

Algorithm	Indices utilised							
	Age	AST	ALT	ALT/AST	GGT	Platelet count	INR	Total cholesterol
**APRI**		x				x		
**CDS**			x			x	x	
**FIB-4**	x	x	x			x		
**Forns’ Index**	x				x	x		x
**GUCI**		x				x	x	
**King’s Score**	x	x				x	x	
**Lok Index**		x	x			x	x	

APRI = AST to Platelet Ratio Index, CDS = Cirrhosis discriminant score, FIB-4 = Fibrosis-4 score, Forns’ = Forns’ Index, GUCI = Göteborg University Cirrhosis Index, King’s = King’s Score, LOK = Lok Index. AST = aspartate aminotransferase, ALT = alanine aminotransferase level, GGT = ɣ-glutamyl transpeptidase, INR = international normalised ratio

### Statistical analysis

The mono-infected cohort were stratified by cirrhosis status and divided into a derivation set (75% of subjects) and a validation set (25% of subjects), by random allocation using the Excel (Microsoft Office Professional Plus 2013 Version) random number generator. The co-infected patients were not included.

For each algorithm, the data from the derivation set were plotted on an XY graph correlating kPa with published cut offs, and the NPV was determined. The number of patients who would not require TE to exclude cirrhosis was determined (“efficiency”). If the derivation cohort NPV was 100%, the algorithm was analysed in the validation cohort. Where the derivation set NPV was less than 100%, two new cut-off thresholds were determined. The first was set at 100% NPV, while the second sought to optimise both NPV and efficiency. The new thresholds were subsequently analysed in the validation set. Efficiency was then determined by calculating the number of TE tests avoided over the total number of subjects. Sub-group analyses were also undertaken (co-infection, gender).

Selected cut-off values (each with greater than 97% NPV and efficiency of greater than 35%) were combined in couplets of algorithms to examine whether combinations of algorithms could further improve the efficiency without significant loss of NPV. Finally, NPV was calculated for combinations of the three best performing algorithms.

Statistical analysis was performed using GraphPad PRISM version 6.04 for Windows (GraphPad, California, USA). Descriptive analysis and the chi-square test were undertaken as appropriate. 95% confidence intervals were calculated for NPV and efficiency.

## Results

Data for 903 patients were referred for analysis. Seventeen duplicates and 34 patients with an IQR of more than 21% in the TE kPa measurement were excluded. A further 3 patients were excluded due to incorrect initial data recording (one patient did not have HCV viraemia, one patient’s TE result was more than 1 year from available laboratory results, and one patient had acute HCV). The final study cohort of 850 subjects included 780 patients with mono-infection and 70 co-infected patients. Thirty-five patients (4%) had missing INR data, 202 (24%) had missing cholesterol data and one patient was on warfarin therapy. These subjects were excluded from analyses of algorithms requiring those data points. One transgender participant was excluded from the gender-based sub-analysis.

Patient characteristics at the time of assessment are shown in **Table 2.** The proportion of women in this study was similar to published Australian data [3, 30]. The overall prevalence of cirrhosis was 16%, which is similar to previous studies [15, 19, 25], although it was significantly higher at the tertiary referral centre (27%) compared with the prison cohort (12%), (chi-square statistic = 22.99; p = < 0.0001). The co-infected cohort was predominantly recruited through the public sexual health clinic, and were almost exclusively male.

**Table 2:** Characteristics of subjects with HCV mono-infection and HCV co-infection.

	Derivation cohort N = 588, n (%)	Validation cohort N = 192, n (%)	Total mono-infected cohort N = 780, n (%)	Co-infected cohort N = 70, n (%)
**Site**				
Prison service	424 (72.1%)	138 (71.9%)	562 (72.1%)	10 (14.3%)
Hospital	150 (25.5%)	52 (27.1%)	202 (25.9%)	5 (7.1%)
Sexual Health clinic	14 (2.4%)	2 (1.0%)	16 (2.0%)	55 (78.6%)
**Gender**				
Male	456 (77.6%)	156 (81.3%)	612 (78.5%)	65 (92.9%)
Female	131 (22.3%)	36 (18.8%)	167 (21.4%)	5 (7.1%)
Transgender	1 (0.2%)	0 (0%)	1 (0.1%)	0 (0%)
**Age**				
Yrs median (range)	41 (18–83)	41 (20–76)	41 (18–83)	44 (26–73)
**Genotype**				
1a/b	312 (53.1%)	113 (58.9%)	425 (54.5%)	43 (61.4%)
2	14 (2.4%)	8 (4.2%)	22 (2.8%)	2 (2.9%)
3	237 (40.3%)	66 (34.4%)	303 (38.8%)	20 (28.6%)
4	10 (1.7%)	1 (0.5%)	11 (1.4%)	2 (2.9%)
6	2 (0.3%)	0 (0%)	2 (0.3%)	1 (1.4%)
Mixed	7 (1.2%)	3 (1.6%)	10 (1.3%)	0 (0%)
Unknown	6 (1.0%)	1 (0.5%)	7 (0.9%)	2 (2.9%)
**Cirrhosis (total)**	94 (16.0%)	31 (16.1%)	125 (16.0%)	15 (21.4%)
Prison Service	49/424 (11.6%)	20/138 (14.5%)	69/562 (12.3%)	6/10 (60%)
Hospital	44/150 (29.3%)	10/52 (19.2%)	54/202 (26.7%)	0/5 (0%)

N = total number

**Fig 1:**
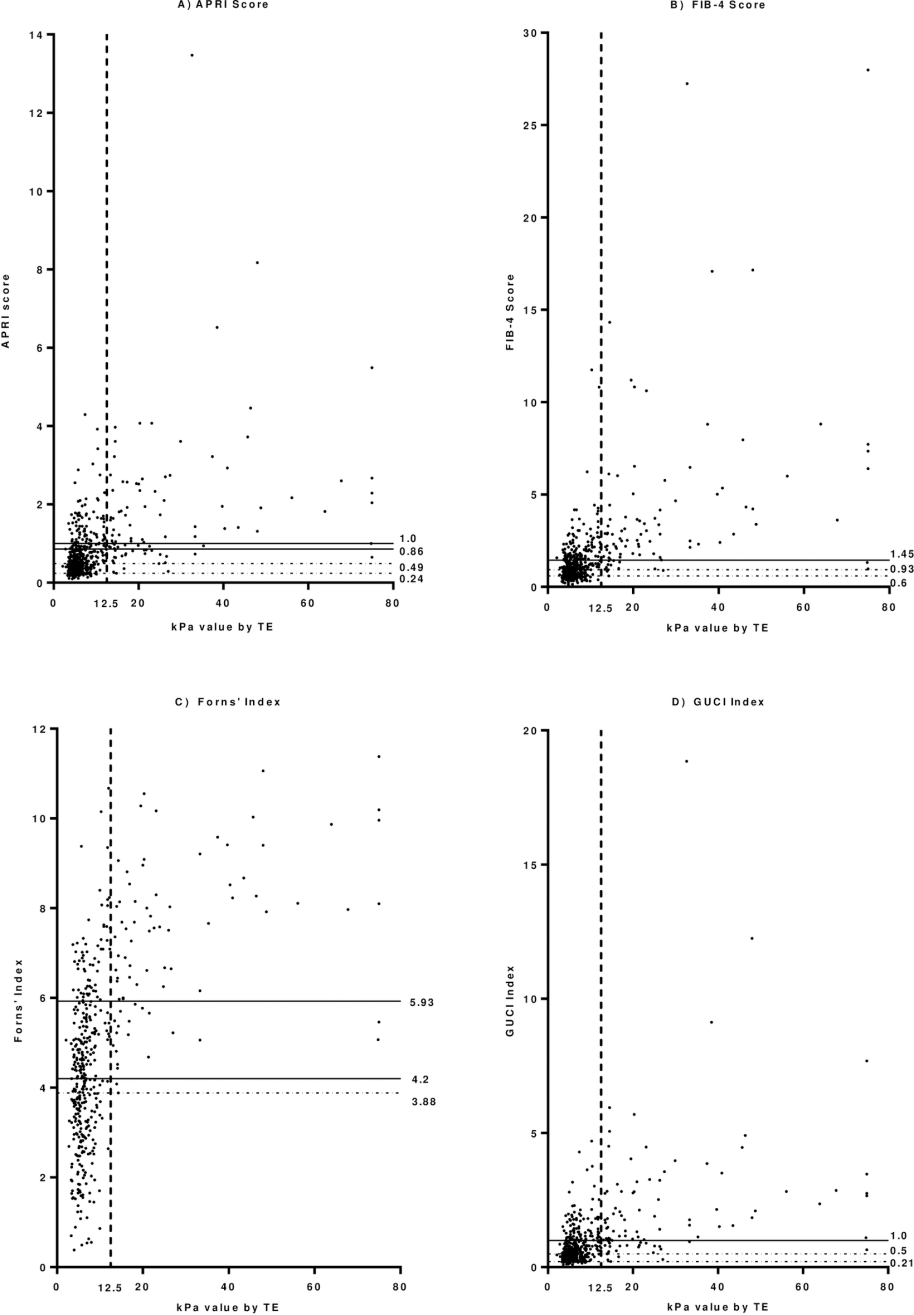
**Hepatitis C Mono-infected derivation cohort showing A) APRI, B) FIB-4, C) Forns’ & D) GUCI scores with published and newly derived cut-off values compared with cirrhosis.** Solid horizontal lines indicate published cut-off values. Dashed horizontal lines indicate newly derived cut-off values. Dashed vertical line indicates TE-defined cirrhosis. TE = transient elastography, kPa = kilopascal.

**Fig 2:**
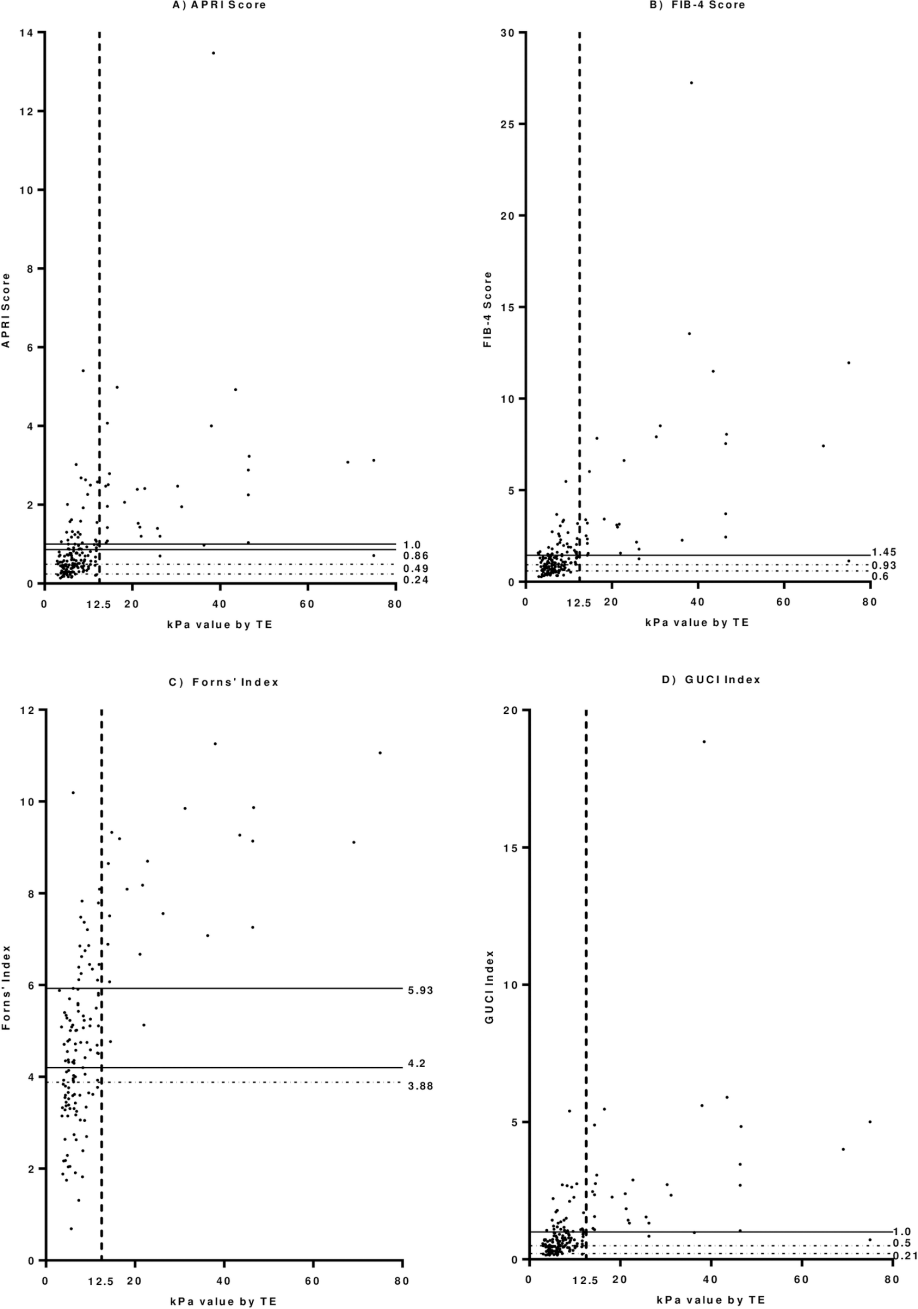
**Hepatitis C Mono-infected validation cohort showing A) APRI, B) FIB-4, C) Forns’ & D) GUCI scores with published and newly derived cut-off values compared with cirrhosis.** Solid horizontal lines indicate published cut-off values. Dashed horizontal lines indicate newly derived cut-off values. Dashed vertical line indicates TE-defined cirrhosis. TE = transient elastography, kPa = kilopascal.

The results from the derivation cohort **(Fig 1)** illustrate that the NPV did not reach 100% in any published algorithm threshold aside from the Forns’ Index. Therefore new cut-offs were calculated. The algorithms performed similarly in the validation cohort (**Fig 2)**. The newly derived cut-offs for APRI (0.49), FIB-4 (0.93) and GUCI (0.5) all had NPVs of 99% (95% CI: 97–100%), allowing reliable avoidance of TE in 40% (315/780), 40% (310/780) and 40% (298/749) of patients respectively **(Table 3).** The NPV of FIB-4 was 100% in the co-infected cohort with reasonable efficiency. The algorithms performed similarly in women and men although the 95% confidence intervals were wider in the female sample. Using combinations of algorithms which performed well revealed that the efficiency could be further improved to above 50% with little reduction in NPV **(Table 4**). All remained more accurate in excluding cirrhosis than the APRI cut-off recommended in the current Australian consensus statement [4].

**Table 3:** Performance of non-invasive fibrosis algorithms in excluding cirrhosis in people with HCV infection.

Algorithms	Cut off	Mono-infection Derivation Cohort NPV% (95% CI), N	Mono-infection Validation Cohort NPV% (95% CI), N	Mono-infection Men NPV% (95% CI), TE avoided/N (%)	Mono-infection Women NPV% (95% CI), TE avoided/N (%)	Mono-infection ALL subjects NPV% (95% CI), TE avoided/N (%)	Co-infection ALL subjects NPV% (95% CI), TE avoided/N (%)
**APRI**	1.0	___	___	94% (91–96%), 415/612 (67.8%)	93% (88–97%), 127/167 (76.0%)	94% (91–96%), 542/780 (69.5%)	98% (88–100%), 45/70 (64.3%)
	0.86	___	___	95% (93–97%), 379/612 (61.9%)	94% (88–97%), 122/167 (73.1%)	95% (93–97%), 501/780 (64.2%)	98% (87–100%), 40/70 (57.1%)
	0.49	98% (96–100%), 588	100% (95–100%), 192	99% (97–100%), 232/612 (37.9%)	98% (92–100%), 83/167 (49.7%)	99% (97–100%), 315/780 (40.4%)	97% (83–100%), 29/70 (41.4%)
	0.24	100% (92–100%), 588	100% (77–100%), 192	100% (91–100%), 41/612 (6.7%)	100% (83–100%), 20/167 (12.0%)	100% (94–100%), 61/780 (7.8%)	100% (54–100%), 6/70 (8.6%)
**CDS**	< 7	___	___	88% (84–90%), 477/586 (81.4%)	90% (84–94%), 133/163 (81.6%)	88% (85–90%), 609/749 (81.3%)	84% (71–92%), 46/64 (71.9%)
	< 4	98% (95–99%), 561	95% (87–99%), 188	97% (94–99%), 233/586 (39.8%)	97% (91–100%), 75/163 (46.0%)	97% (94–98%), 308/749 (41.1%)	95% (76–100%), 20/64 (31.3%)
	< 3	100% (97–100%), 561	97% (86–100%), 188	99% (95–100%), 111/586 (18.9%)	100% (91–100%), 41/163 (25.2%)	99% (96–100%), 152/749 (20.3%)	100% (59–100%), 7/64 (10.9%)
**FIB-4**	1.45	___	___	97% (94–98%), 395/612 (64.5%)	97% (92–99%), 99/167 (59.3%)	97% (95–98%), 494/780 (63.3%)	90% (76–97%), 36/70 (50.4%)
	0.93	99% (96–100%), 588	100% (95–100%), 192	99% (97–100%), 249/612 (40.7%)	98% (91–100%), 61/167 (36.5%)	99% (97–100%), 310/780 (39.7%)	100% (84–100%), 21/70 (30.0%)
	0.6	100% (96–100%), 588	100% (88–100%), 192	100% (96–100%), 103/612 (16.8%)	100% (87–100%), 26/167 (15.6%)	100% (97–100%), 129/780 (16.5%)	100% (16–100%), 2/70 (2.9%)
**Forns’**	5.93	___	___	94% (91–97%), 318/459 (69.3%)	97% (91–99%), 90/132 (68.2%)	95% (92–97%), 408/592 (68.9%)	95% (82–99%), 35/55 (63.6%)
	4.2	___	___	99% (97–100%), 185/459 (40.3%)	98% (90–100%), 53/132 (40.2%)	99% (97–100%), 238/592 (45.3%)	100% (79–100%), 16/55 (29.1%)
	3.88	100% (98–100%), 451	100% (92–100%), 141	100% (98–100%), 159/459 (34.6%)	100% (91–100%), 41/132 (31.1%)	100% (98–100%), 200/592 (33.8%)	100% (75–100%), 13/55 (23.6%)
**GUCI**	1.0	___	___	95% (92–97%), 382/586 (65.2%)	94% (88–97%), 121/163 (74.2%)	95% (92–96%), 503/749 (67.2%)	97% (87–100%), 38/64 (59.4%)
	0.5	98% (96–100%), 561	100% (95–100%), 188	99% (97–100%), 213/586 (48.3%)	98% (92–100%), 85/163 (52.1%)	99% (97–100%), 298/749 (39.8%)	96% (81–100%), 26/64 (40.6%)
	0.21	100% (85–100%), 561	100% (69–100%), 188	100% (83–100%), 20/586 (3.4%)	100% (74–100%), 12/163 (7.4%)	100% (89–100%), 32/749 (4.3%)	100% (2.5–100%), 1/64 (1.6%)
**King’s**	16.7	___	___	96% (94–98%), 387/586 (66.0%)	95% (90–98%), 121/163 (74.2%)	96% (94–98%), 508/749 (67.8%)	97% (86–100%), 37/64 (57.8%)
	8.7	98% (95–99%), 561	100% (95–100%), 188	99% (97–100%), 243/586 (41.5%)	96% (90–99%), 80/163 (49.1%)	98% (96–100%), 323/749 (43.1%)	100% (85–100%, 23/64 (35.9%)
	5.46	100% (97–100%), 561	100% (92–100%), 188	100% (97–100%), 129/586 (22.0%)	100% (92–100%), 44/163 (27.0%)	100% (98–100%), 173/749 (23.1%)	100% (74–100%), 12/64 (18.8%)
**LOK**	0.2	___	___	96% (93–98%), 206/586 (35.2%)	95% (88–99%), 79/163 (48.5%)	96% (93–98%), 285/749 (38.1%)	93% (76–99%), 25/64 (39.1%)
	0.168	98% (94–99%), 561	96% (86–100%), 188	98% (94–100%), 152/586 (25.9%)	96% (87–99%), 64/163 (39.3%)	97% (94–99%), 216/749 (28.8%)	89% (67–99%), 17/64 (26.6%)
	0.109	100% (95–100%), 561	96% (80–100%), 188	98% (91–100%), 60/586 (10.2%)	100% (91–100%), 38/163 (23.3%)	99% (95–100%), 98/749 (13.1%)	100% (48–100%), 5/64 (7.8%)

NPV = negative predictive value, CI = confidence interval, N = total number, TE = transient elastography. APRI = AST to Platelet Ratio Index, CDS = Cirrhosis discriminant score, FIB-4 = Fibrosis-4 score, Forns’ = Forns’ Index, GUCI = Göteborg University Cirrhosis Index, King’s = King’s Score, LOK = Lok Index.

**Table 4:** Performance of combinations of non-invasive fibrosis algorithms in excluding cirrhosis in people with HCV infection.

Algorithms with cut-off values	Mono-infection ALL subjects NPV% (95% CI), TE avoided/N (%)
**APRI <0.49 or FIB-4 <0.93**	99% (97–99%), 418/780 (53.6%)
**APRI <0.49 or GUCI <0.5**	99% (97–99%), 319/749 (42.6%)
**FIB-4 <0.93 or GUCI <0.5**	98% (97–99%), 398/749 (53.1%)
**APRI <0.49 or FIB-4 <0.93 or GUCI < 0.5**	99% (97–99%), 405/749 (54.1%)

NPV = negative predictive value, CI = confidence interval, TE = transient elastography, N = total number. APRI = AST to Platelet Ratio Index, FIB-4 = Fibrosis-4 score, GUCI = Göteborg University Cirrhosis Index.

## Discussion

This study demonstrated the potential to significantly improve the efficiency of HCV assessment by replacing the use of TE with non-invasive fibrosis algorithms in approximately 50% of cases. This, in turn, will enable primary care physicians to assess and treat patients without the need to refer to specialist care in many cases, enhancing the probability of achieving the WHO HCV elimination goals [1].

The strengths of this study include the large sample size, the multi-centre recruitment, as well as the proportion of cirrhotic patients included in the study. Limitations include: the partially retrospective design; that only 3 sites were included; and that men were over-represented, although no differences were identified by gender. The study findings are consistent with other reports [14], including recent Australian data, which also found a NPV of only 94% in a cohort of 677 patients when utilising APRI with a cut off of 1.0 [25].

Both American and European guidelines accept use of either TE or algorithms in assessment, while Australian guidelines emphasise TE, with non-invasive algorithms suggested for cirrhosis evaluation if TE is not accessible in a timely fashion [4, 10, 31]. APRI, with a cut-off of 1.0 is quoted, although this only reached 94% NPV (95% CI: 91–96%) in this study, meaning that a small but significant population would not be identified as having cirrhosis and would miss out appropriate screening for HCC and other complications of cirrhosis. Revising the APRI cut-off to 0.49 allowed for an increase in NPV to 99% (95% CI: 97–100%), while still retaining adequate utility.

Most algorithms were developed to positively identify cirrhosis, rather than reliably rule out advanced liver disease. Despite this, the positive predictive value (PPV) for many of these algorithms is poor, including the original published data for APRI (cut off 1.0; PPV 38%), King’s (cut off 16.7; PPV 56%) and GUCI (cut off 1.0; PPV 31%) [15, 20–21]. Interestingly, the Forns’ Index which was devised to exclude fibrosis, was the only algorithm which performed well, with a high NPV and good efficiency based on the original published data [19]. While not necessarily a barrier to its use, the Forns’ Index requires total cholesterol in addition to other routine blood tests, which is an additional cost.

Based on these findings it is reasonable to propose an updated approach to the assessment of HCV in services that do not have immediate access to TE. An APRI of <0.49, a FIB-4 of <0.93 or a GUCI score of <0.5 can reliably exclude cirrhosis and the need for TE. If the patient does not meet the threshold in one algorithm then the others can be performed, and if the patient is below the relevant threshold in any of the three algorithms then cirrhosis can be considered to be excluded. This approach could be implemented cheaply and safely, as it requires only routine blood tests such as platelet count, liver function tests and INR. Importantly, the algorithms are easy to calculate in the primary care setting with readily accessible online calculators [32]. If cirrhosis is not excluded by the non-invasive algorithms then the patient should proceed to TE for fibrosis assessment. The use of algorithms in combination has been evaluated in a small number of studies with concordant results to those described here [25, 33]. Specifically, Bloom *et al* combined APRI with a cut off of 0.853 and FIB-4 with a cut off of 1.531 in 677 patients to reach an NPV of 96.6% [25].

Although DAA treatments with similar efficacy in cirrhotic and non-cirrhotic patients are now available in Australia, there remains an ongoing increased risk of mortality due to HCC and clinical disease progression in people with advanced fibrosis and cirrhosis despite cure of HCV infection. This makes identification of cirrhosis and follow-up of this population an important element of clinical care [7–10].

With universal health care ensuring access to testing and subsidised DAA treatment, Australia is poised as a global leader in HCV elimination. A further 200,000 people or almost 1% of the population still require treatment for chronic HCV. Hence, streamlining and simplifying the assessment process in a significant proportion of patients will assist in improving the care cascade, particularly in the primary care setting. This study confirms that readily available clinical algorithms can aid clinicians to confidently rule out cirrhosis, and move on to more readily prescribe, treat and cure chronic HCV.

## Supporting information

S1 Table Anonymised data set. HCV = Hepatitis C Virus, HBV = Hepatitis B Virus, HIV = Human immunodeficiency virus, kPa = kilopascal, IQR = Interquartile range, AST = aspartate aminotransferase, AST ULN = aspartate aminotransferase upper limit of normal, ALT = alanine aminotransferase level, GGT = ɣ-glutamyl transpeptidase, INR = international normalised ratio. Site information has been anonymised.
(PDF)
