# Supplementary material for: Capacity of non-invasive hepatic fibrosis algorithms to replace transient elastography to exclude cirrhosis in people with hepatitis C virus infection: A multi-centre observational study
Source: PLoS One. 2018 Feb 13;13(2):e0192763. doi: 10.1371/journal.pone.0192763 (PMC5811020; doi:10.1371/journal.pone.0192763)
Supplement: S1 Table — HCV = Hepatitis C Virus, HBV = Hepatitis B Virus, HIV = Human immunodeficiency virus, kPa = kilopascal, IQR = Interquartile range, AST = aspartate aminotransferase, AST ULN = aspartate aminotransferase upper limit of normal, ALT = alanine aminotransferase level, GGT = ɣ-glutamyl transpeptidase, INR = international normalised ratio. Site information has been anonymised. (PDF) [file pone.0192763.s001.pdf]

Unique identifier (site anonymised)	Age (years)	Sex (1 = Male, 2 = Female)	HCV mono-infection	Co-infected with HBV (1), HIV (2), both (3)?	Genotype	kPa	IQR (%)	AST	AST ULN	ALT	GGT	Platelets	INR	Cholesterol
1	55	1t	1	0	1a	10	6	61	40	73	230	125	1.1	4.5
2	43	2	1	0	3a	8.8	5	112	35	214	52	248	0.9	X
3	37	2	0	2	3a	8.2	17	48	45	61	21	247	1.3	3.1
4	36	2	0	2	3a	4.3	7	30	45	56	33	177	0.9	4.9
5	53	2	1	0	1a	8.9	16	67	45	83	98	190	X	4.5
6	33	2	0	2	4	3.4	6	42	45	46	36	256	1.0	4.7
7	39	2	1	0	1a	5.6	13	49	45	59	368	278	1.1	5.6
8	24	2	1	0	1a	7.0	14	33	45	77	26	388	1.1	4.0
9	40	2	1	0	1a	10.0	11	125	45	196	33	285	1.2	2.9
10	76	2	1	0	1a	46.4	2	129	35	103	103	128	1.2	3.9
11	54	2	1	0	3a	19.5	14	50	45	30	69	44	1.6	5.2
12	22	2	1	0	1a	8	9	20	45	25	12	163	1.0	4.6
13	55	2	1	0	3a	8.8	15	109	45	199	31	126	1.1	4.6
14	61	2	1	0	1a	3.9	10	59	45	75	22	223	1.0	4.6
15	49	2	1	0	1a	4.6	9	45	45	58	17	222	1.1	4.6
16	53	2	1	0	1a	9.5	12	103	45	179	307	182	1.1	4.8
17	58	2	1	0	2b	6.8	13	26	45	29	18	179	1.0	4.7
18	58	2	1	0	1a	9	15	28	45	23	28	299	1.0	4.4
19	61	2	1	0	3a	75	0	84	45	29	27	34	1.4	4.1
20	64	2	1	0	1a	14.6	14	62	45	69	89	131	1.0	4.3
21	60	2	1	0	1a	4.7	15	33	45	39	83	233	0.9	4.4
22	60	2	1	0	2c	20.3	9	114	45	94	159	108	1.2	4.1
23	33	2	1	0	1a	7	12	62	45	42	10	312	1.1	4.9
24	60	2	1	0	3a	63.9	15	54	45	31	49	66	1.3	3.8
25	57	2	1	0	3a	8.8	10	68	45	55	124	335	1.0	4.6
26	60	2	1	0	1a	2.1	10	38	45	51	16	202	1.1	4.6
27	54	2	1	0	3a	8.8	10	21	45	14	12	276	1.0	5.3

28	57	2	1	0	3a	22.6	14	63	45	49	150	150	1.1	4.6
29	58	2	1	0	1a	6.3	10	38	45	34	11	229	1.1	4.5
30	24	2	1	0	3a	3.8	14	55	45	100	11	191	1.1	5.0
31	60	2	1	0	4	5.6	11	20	45	24	11	235	1.0	4.4
32	59	2	1	0	1b	9.4	13	85	45	120	156	236	1.0	5.1
33	54	2	1	0	1b	24.8	18	49	45	44	156	221	1.1	4.5
34	56	2	1	0	3a	8.4	8	43	45	54	25	220	1.0	4.3
35	45	2	1	0	1a	14.1	16	32	45	21	69	260	1.0	4.4
36	54	2	1	0	1a	5.9	14	63	45	101	33	321	1.0	4.3
37	45	2	1	0	3a	2.9	21	54	45	63	33	297	1.0	4.9
38	42	2	1	0	1b	4.4	10	34	45	44	23	331	1.0	5.3
39	45	2	1	0	3a	9.4	9	171	45	226	138	210	1.0	2.8
40	61	2	1	0	1a	21.5	14	170	45	222	235	195	1.1	4.3
41	53	2	1	0	1a	4.8	12	47	45	54	18	158	1.0	5.8
42	46	2	1	0	3a	4.9	15	47	45	73	22	316	1.0	4.3
43	55	2	1	0	3a	37.4	13	113	45	82	104	78	1.2	3.9
44	55	2	1	0	1a	5.2	9	48	45	65	30	180	1.0	4.1
45	61	2	1	0	1b	6.9	10	35	45	32	22	242	1.0	4.2
46	54	2	1	0	1a	4.9	9	32	45	37	24	236	1.0	4.0
47	50	2	1	0	1a	4.1	11	44	45	68	21	212	1.0	4.2
48	41	2	1	0	3a	5.6	12	63	45	103	67	231	1.0	4.8
49	67	2	1	0	4	21.8	10	74	45	79	138	196	1.1	3.5
50	63	2	1	0	1b	31.2	12	64	45	42	71	73	1.2	4.1
51	49	2	1	0	4	4.3	11	26	45	30	9	279	1.0	3.9
52	54	2	1	0	3a	7.9	15	43	45	34	52	186	1.0	3.9
53	57	2	1	0	1a	4	11	85	45	54	115	321	1.0	5.1
54	63	2	1	0	3a	8.9	16	35	45	36	21	187	1.0	4.7
55	56	2	1	0	1a	9.3	12	228	45	146	100	193	1.0	3.1
56	47	2	1	0	1b	7.3	10	72	45	105	46	205	1.1	3.8
57	55	2	1	0	1b	5.8	17	38	45	52	46	229	1.0	4.6
58	51	2	1	0	2b	7.9	10	29	45	41	35	245	1.0	5.6
59	79	2	1	0	1a	12	12	63	45	57	83	61	1.1	5.3
60	41	2	1	0	1b	4.6	19	80	45	92	60	212	1.0	5.6

61	46	2	1	0	1a	6.9	17	23	45	28	27	231	0.9	4.9
62	67	2	1	0	4	16.1	12	80	45	95	413	263	0.9	3.9
63	64	2	1	0	1a	14.1	16	115	45	139	31	189	1.1	3.8
64	43	2	1	0	1a	7.6	15	24	45	19	104	248	1.0	4.0
65	53	2	1	0	1a	5.4	18	30	45	24	25	267	0.9	5.3
66	58	2	1	0	1a	5.1	18	38	45	38	18	237	0.9	5.0
67	59	2	1	0	1b	4	16	31	45	43	15	167	1.0	4.8
68	53	2	1	0	1b	6.2	19	38	45	43	45	288	0.9	5.4
69	41	2	1	0	1b	6.3	17	25	45	30	41	244	1.1	4.3
70	62	2	1	0	1a	20.9	9	69	45	71	94	139	1.2	4.1
71	51	2	1	0	3a	10.9	19	193	45	203	197	156	1.1	4.0
72	43	2	1	0	3a	4.8	16	53	45	97	78	244	1.0	4.6
73	53	2	1	0	3a	6.2	17	132	45	143	36	140	1.1	5.2
74	45	2	1	0	1b	7.7	6	54	45	82	51	283	0.9	4.1
75	56	2	1	0	1b	9.9	14	82	45	90	39	255	1.0	4.0
76	63	2	1	0	1b	4.4	8	59	45	45	79	238	0.9	4.9
77	59	2	1	0	1a	11.8	6	92	45	79	200	143	1.0	4.6
78	40	2	1	0	1a	4.4	5	56	45	118	26	251	0.9	4.5
79	24	2	1	0	1a	5.7	11	50	45	80	28	377	1.0	4.0
80	59	2	1	0	1b	6.7	10	46	45	43	25	198	1.0	3.8
81	32	2	1	0	3a	33.3	7	77	45	68	152	120	1.1	4.8
82	60	2	1	0	1b	14.8	12	22	45	29	23	207	0.9	3.8
83	31	2	1	0	3a	5.5	9	112	45	178	36	227	1.0	4.3
84	53	2	1	0	1b	5.5	3	27	45	27	18	201	1.0	4.1
85	59	2	1	0	1a	5.7	4	47	45	44	17	260	0.9	4.7
86	30	2	1	0	3a	5.8	10	35	45	90	32	234	0.9	4.2
87	62	2	1	0	1a	7.1	11	94	45	142	27	160	1.0	4.7
88	48	2	1	0	2b	8.6	6	30	45	26	41	86	1.0	5.2
89	60	2	1	0	1a	6.6	6	22	45	29	23	207	0.9	4.6
90	59	2	1	0	1a	6.9	3	57	45	76	102	231	0.9	5.1
91	58	2	1	0	1a	5.3	6	42	45	38	30	227	1.0	4.1
92	57	2	1	0	4	8.8	9	25	45	27	34	164	1.2	3.2
93	57	2	1	0	1a	13.9	8	78	45	82	286	171	1.0	3.9

94	48	2	1	0	3a	6.9	12	40	35	42	29	307	1.0	X
95	48	2	1	0	1a	6.9	7	34	35	45	70	258	1.0	X
96	36	2	1	0	1a	3.9	15	28	35	33	46	253	1.0	3.8
97	57	2	1	0	3a	7.4	8	126	35	168	93	181	1.0	2.5
98	41	2	1	0	1a	3.3	21	24	35	33	25	338	1.0	5.6
99	33	2	1	0	3a	8.6	6	93	35	95	53	341	0.9	3.9
100	39	2	1	0	1a	4	10	39	35	54	52	339	1.0	3.7
101	38	2	1	0	3a	3.6	17	18	35	34	41	474	0.9	4.3
102	39	2	1	0	1a	11.6	10	47	35	48	86	235	1.2	3.0
103	32	2	1	0	1a	4.9	18	34	35	58	22	282	0.9	5.4
104	18	2	1	0	2b	4.8	17	40	35	111	26	256	X	3.3
105	37	2	1	0	1b	5.4	9	17	35	25	16	289	1.0	5.0
106	22	2	1	0	1a	3.4	15	35	35	65	31	230	1.0	3.6
107	21	2	1	0	3a	4	8	32	35	76	27	362	1.0	3.9
108	34	2	1	0	1a	6.4	9	37	35	42	58	250	1.0	3.5
109	31	2	1	0	1a	5.3	4	28	35	41	16	198	1.0	5.6
110	35	2	1	0	3a	6.5	9	19	35	42	17	171	1.0	4.6
111	32	2	1	0	3a	4.3	7	20	35	37	35	305	0.9	3.8
112	28	2	1	0	3a	5.4	7	44	40	58	25	251	1.0	X
113	31	2	1	0	3a	7.6	11	31	35	45	32	172	0.9	X
114	53	2	1	0	3a	4.8	8	27	35	38	15	310	0.9	X
115	27	2	1	0	3a	5.6	9	47	35	73	59	315	1.0	X
116	32	2	1	0	3a	6.1	3	59	35	124	180	191	1.0	6.3
117	46	2	1	0	3a	7.8	9	39	35	50	37	236	1.1	4.2
118	43	2	1	0	1b	6	7	25	35	41	106	238	0.9	X
119	38	2	1	0	3a	6.7	6	93	35	152	119	312	1.0	6.1
120	41	2	1	0	1a	4.5	10	100	35	288	142	258	1.0	X
121	30	2	1	0	3a	5.7	18	37	35	63	41	175	1.0	3.7
122	34	2	1	0	3a	5	10	44	35	71	21	243	1.0	X
123	42	2	1	0	1b	14.3	8	63	35	98	144	127	1.1	X
124	45	2	1	0	3a	3.6	17	39	35	45	25	250	1.0	5.7
125	39	2	1	0	1a	13.9	14	42	35	81	63	214	0.9	5.3
126	35	2	1	0	1a	4.4	18	46	35	94	35	227	0.9	4.3

127	44	2	1	0	3a	5.6	16	68	35	102	36	272	1.0	X
128	37	2	1	0	1a	4.5	13	15	35	21	19	254	1.0	X
129	50	2	1	0	1a	5.4	7	40	35	79	69	250	0.9	X
130	46	2	1	0	3a	6.3	6	46	35	86	38	242	1.0	4.8
131	28	2	1	0	3a	6	10	37	35	60	36	214	0.9	4.7
132	45	2	1	0	3a	6.3	13	35	35	46	59	334	0.9	X
133	43	2	0	2	3a	13.8	12	105	35	153	61	262	1.0	2.4
134	57	2	1	0	1b	7.4	4	49	35	55	89	185	0.9	4.8
135	44	2	1	0	1a	5.6	16	30	35	30	126	152	0.9	X
136	27	2	1	0	3a	3	10	30	35	56	18	382	0.9	X
137	40	2	1	0	1a	13.5	7	87	35	114	149	150	1.0	2.7
138	30	2	1	0	3a	4.6	11	27	35	69	32	220	X	3.5
139	43	2	1	0	3a	4.3	7	41	35	66	27	260	1.0	X
140	25	2	1	0	1a	5.8	7	15	35	47	42	385	0.9	X
141	50	2	1	0	2	5.7	14	28	35	32	36	198	1.0	4.8
142	36	2	1	0	3a	11.5	15	67	35	80	169	248	0.9	X
143	51	2	1	0	3a	12	2	36	35	44	33	103	1.0	X
144	38	2	1	0	1b	16.9	8	46	35	35	117	184	1.1	2.5
145	36	2	1	0	3a	8.9	6	77	35	185	60	231	1.1	2.7
146	40	2	1	0	3a	4.4	6	42	35	53	28	259	0.9	X
147	29	2	1	0	3a	3.9	7	72	35	130	50	236	1.0	X
148	47	2	1	0	2b	3.3	2	38	35	63	17	136	0.9	X
149	43	2	1	0	3a	9.2	3	29	35	48	26	187	1.0	
150	28	2	1	0	3a	5.6	8	41	35	96	42	232	0.9	4.5
151	32	2	1	0	1a	4.8	7	11	35	42	33	270	1.0	X
152	38	2	1	0	1a	4.5	9	32	35	45	74	253	0.9	X
153	18	2	1	0	1b	4.8	3	30	35	154	103	367	1.0	X
154	30	2	1	0	1a	3.8	11	38	35	55	20	140	X	X
155	32	2	1	0	1a	3.9	15	27	35	50	36	229	1.1	5.1
156	30	2	1	0	3a	4.7	4	55	35	102	50	264	1.0	X
157	35	2	1	0	3a	7.7	12	90	35	173	31	212	1.0	X
158	30	2	1	0	3a	2.8	11	79	35	194	42	263	1.0	4.4
159	37	2	1	0	3a	4.7	9	60	35	67	55	348	0.9	X

160	31	2	1	0	1a	6.2	5	84	35	188	96	357	1.0	X
161	30	2	1	0	1a	3.6	3	27	35	49	23	295	0.9	4.3
162	39	2	1	0	1a	4	15	19	35	16	22	313	1.0	5.1
163	38	2	1	0	1a	6.3	11	33	35	44	48	355	0.9	4.6
164	34	2	1	0	unknown	4.8	2	56	35	60	69	223	0.9	6.0
165	41	2	1	0	1a	5.3	6	40	35	31	35	284	1.0	4.0
166	47	2	1	0	2	6	8	48	35	73	27	181	1.1	4.3
167	45	2	1	0	3a	5	14	42	35	56	33	302	0.9	X
168	59	2	1	0	2a & 2c	9.5	6	37	35	27	39	279	1.0	X
169	34	2	1	0	3a	5.3	6	23	35	33	23	290	1.0	5.6
170	50	2	1	0	3a	12.5	7	75	35	64	498	297	1.0	3.7
171	56	2	0	2	1b	6.1	7	48	45	35	40	171	1.1	4.5
172	58	2	1	0	1a	18	8	120	45	149	87	181	1.2	2.6
173	45	2	1	0	1a	6.1	11	19	45	20	205	70	1.1	3.1
174	39	1	0	2	1a	6.1	8	29	45	38	27	221	1.0	6.3
175	38	1	0	2	1a	5.1	16	41	45	66	58	198	1.0	4.4
176	43	1	0	2	1a	4.0	18	52	45	77	86	169	1.0	3.2
177	33	1	0	2	1a	4.3	14	33	45	68	33	150	0.9	4.4
178	50	1	0	2	1a	5.3	13	102	45	142	108	213	X	5.1
179	38	1	0	2	1a	20.4	7	293	45	265	282	129	1.2	3.3
180	54	1	0	2		4.4	18	49	45	48	131	286	1.1	4.7
181	39	1	0	2	1a	6.0	12	59	45	70	186	273	X	5.5
182	33	1	0	2	1a	7.3	21	60	45	93	39	259	1.1	4.4
183	47	1	0	2	1a	6.7	12	91	45	427	388	169	1.0	3.6
184	55	1	0	2	1a	6.4	14	24	45	35	55	212	0.9	3.8
185	63	1	0	2	1a	7.1	14	126	45	99	162	117	0.9	X
186	46	1	0	2	1a	5.3	15	56	45	128	59	286	1.1	3.7
187	38	1	0	2	1a	3.6	3	60	45	95	84	264	1.0	3.2
188	37	1	0	2	3a	7.3	10	150	45	115	155	252	0.9	4.6
189	38	1	0	2	1a	5.3	2	85	45	132	320	259	0.9	3.6
190	51	1	0	2	1a	4.3	9	66	45	186	31	220	1.0	3.0
191	26	1	0	2	1a	5.9	14	38	45	65	18	217	1.0	4.1
192	43	1	0	2	1a	4.3	7	38	45	55	37	340	1.0	5.6

193	73	1	1	0	1b & 3a	11.6	6	84	45	80	31	173	1.0	X
194	32	1	0	2	3a	26.0	21	107	45	168	96	218	1.1	X
195	44	1	0	2	6	4.3	2	24	45	31	19	282	X	3.6
196	43	1	1	0	1b	75.0	0	70	45	147	60	218	1.0	X
197	73	1	0	3	3a	12.3	10	35	45	35	24	191	1.0	3.7
198	62	1	0	2	1a	7.1	17	54	45	66	35	293	1.0	4.0
199	48	1	0	2	1a	4.8	10	38	45	33	50	210	0.9	5.0
200	45	1	1	0	3a	4.8	15	59	45	79	42	174	1.3	1.9
201	52	1	0	2	3a	16.5	15	135	40	94	495	99	1.0	3.4
202	49	1	0	2	1a	6.2	11	26	45	20	55	140	1.1	3.4
203	40	1	1	0	1a	11.8	8	84	45	151	122	206	1.0	3.5
204	35	1	0	2	1a	7.1	14	34	45	60	75	338	1.0	X
205	64	1	0	2	3a	4.3	5	29	45	29	34	189	1.1	X
206	33	1	0	2	1a	7.3	11	114	45	189	437	236	X	5.9
207	34	1	0	2	1a	6.7	13	34	45	54	67	231	1.0	5.8
208	34	1	0	2	3a	8.1	5	48	45	121	54	209	1.0	X
209	52	1	0	2	1a	4.0	15	20	41	27	25	236	1.1	X
210	60	1	0	2	1a	3.7	11	73	45	76	568	274	1.0	4.8
211	44	1	0	2	1a	32.4	17	120	45	69	74	112	1.2	X
212	48	1	0	2	3a	11.7	17	127	45	209	121	69	1.4	3.0
213	37	1	0	2	3k	5.8	17	35	45	38	15	251	X	4.3
214	50	1	0	2	3a	7.8	6	69	30	61	21	104	1.0	2.7
215	30	1	0	2	1a	6.6	6	88	45	232	101	268	1.0	X
216	53	1	0	2	1a	6.6	12	21	45	17	19	193	0.9	3.8
217	39	1	1	0	1a	8.8	3	97	36	141	178	288	1.0	3.0
218	56	1	0	2	3a	7.2	21	57	45	92	41	275	1.0	6.0
219	42	1	0	2	1b	25.1	16	103	45	155	431	205	0.9	6.2
220	53	1	0	2	3a	35.8	11	55	45	20	61	73	1.4	2.5
221	49	1	0	2	1a	4.9	8	24	45	28	38	278	0.9	3.9
222	48	1	0	2	3a	6.6	15	106	45	151	43	246	X	3.2
223	37	1	0	2	1a	4.2	12	47	45	64	73	145	0.9	X
224	39	1	0	2	4	4.5	18	44	45	82	55	252	1.0	4.3
225	46	1	0	2	3a	12.6	5	205	45	235	167	192	1.1	3.9

226	43	1	0	2	1a	42.8	11	78	45	78	143	169	1.1	4.2
227	35	1	0	2	1a	9.1	12	140	45	246	74	217	0.9	4.0
228	43	1	0	2	2	7.1	10	34	45	35	35	334	1.0	5.2
229	32	1	1	0	3a	9.2	9	48	45	103	98	235	1.1	5.0
230	40	1	1	0	3a	8.0	18	62	45	110	16	196	X	X
231	44	1	1	0	1a	18.2	16	98	45	35	975	193	1.2	3.1
232	60	1	1	0	1a	5.7	14	84	45	161	259	109	1.1	4.2
233	68	1	0	2	1a	6.1	8	58	45	68	60	130	1.1	5.2
234	48	1	0	2	1a	9.5	11	101	45	156	91	221	1.0	4.2
235	54	1	1	0	3a	9.7	21	53	45	92	257	249	1.1	3.5
236	25	1	1	0	3a	6.3	10	82	45	146	28	241	1.0	X
237	37	1	1	0	3a	6.3	10	72	45	42	54	266	1.0	4.9
238	47	1	1	0	1b	11.5	9	46	45	84	60	221	1.0	5.1
239	39	1	1	0	3a	4	10	42	45	80	19	210	1.0	4.6
240	59	1	1	0	1a	33.3	11	81	45	82	51	247	1.3	5.0
241	50	1	1	0	1b	5.1	11	55	45	79	23	215	1.1	5.3
242	54	1	1	0	3a	12.1	10	96	45	212	67	188	1.0	5.7
243	51	1	1	0	1a	11.6	9	32	45	25	56	343	0.9	4.4
244	62	1	1	0	1b	12.3	15	56	45	53	174	224	1.1	3.9
245	56	1	1	0	1b	7.3	9	60	45	97	22	155	1.1	4.3
246	53	1	1	0	3a	21.3	6	77	45	82	26	206	0.9	5.1
247	47	1	1	0	1a	7.1	12	60	45	78	29	185	1.0	4.9
248	58	1	1	0	1a	6.1	12	119	45	292	310	184	1.0	5.0
249	51	1	1	0	1a	21.5	9	77	45	96	180	230	1.1	5.2
250	55	1	1	0	1a	5.9	14	99	45	140	106	161	1.0	4.7
251	59	1	1	0	1a	45.7	10	169	45	154	507	101	1.2	4.3
252	49	1	1	0	1b	5.6	6	24	45	22	27	243	1.0	4.2
253	54	1	1	0	1a	15.1	13	43	45	60	31	105	1.1	3.8
254	27	1	1	0	1a	6.4	11	76	45	152	420	331	1.1	4.0
255	52	1	1	0	3a	4.66	7	65	45	111	29	267	1.0	4.5
256	53	1	1	0	1a	19.9	12	102	45	158	133	238	1.1	4.6
257	39	1	1	0	1a	5.4	14	36	45	19	30	291	1.2	4.4
258	24	1	1	0	1b	3.4	12	44	45	99	20	243	1.0	4.3

259	655	1	1	0	1b	20	13	128	45	153	157	113	1.1	3.5
260	70	1	1	0	1b	4.7	6	55	45	37	133	226	1.0	4.0
261	35	1	1	0	1b	5.2	15	24	45	28	27	334	1.0	5.2
262	27	1	1	0	1a	8.7	3	92	45	215	81	255	1.1	4.2
263	42	1	1	0	1a	9.7	14	66	45	56	50	183	1.0	4.3
264	50	1	1	0	1a	5.3	12	57	45	120	24	202	1.1	3.5
265	58	1	1	0	3a	6.8	15	39	45	66	33	198	0.9	4.7
266	48	1	1	0	1b	5.4	6	64	45	155	46	198	1.2	4.5
267	80	1	1	0	1b	10.3	4	134	45	144	56	76	1.2	4.5
268	58	1	1	0	1a	7.9	14	66	45	72	149	267	1.0	4.4
269	58	1	1	0	1a	7.7	18	43	45	77	141	210	1.1	4.2
270	60	1	1	0	1a	3.8	11	30	45	40	34	189	0.9	4.0
271	40	1	1	0	2a	4.9	15	39	45	75	37	196	1.0	3.9
272	50	1	1	0	3a	9	8	64	45	76	75	187	0.9	4.1
273	55	1	1	0	3a	8	12	47	45	75	30	195	1.0	3.0
274	56	1	1	0	1a	7.6	9	32	45	30	22	220	1.0	4.0
275	64	1	1	0	1a	6.1	10	32	45	49	157	225	1.1	4.2
276	52	1	1	0	1a	3.3	12	46	45	106	27	220	1.1	4.1
277	53	1	1	0	1a	8.1	14	86	45	169	79	153	1.2	2.6
278	55	1	1	0	1a	3.7	5	41	45	44	62	147	1.1	3.9
279	36	1	1	0	1b	4.5	12	48	45	76	262	238	1.1	3.9
280	61	1	1	0	3a	12	14	69	45	73	163	155	1.0	4.0
281	51	1	1	0	1a	4.7	16	32	45	58	82	241	1.0	4.5
282	47	1	1	0	1b	13.4	15	150	45	138	70	208	1.1	4.2
283	60	1	1	0	1a	8.9	13	79	45	91	91	229	1.0	5.0
284	55	1	1	0	1a	14	9	87	45	103	759	188	1.1	3.4
285	55	1	1	0	1a	14.3	15	131	45	341	179	269	1.0	4.0
286	35	1	1	0	1a	8.1	9	33	45	42	29	225	0.9	4.3
287	47	1	1	0	3a	12	12	47	45	92	36	190	0.9	4.3
288	60	1	1	0	1b	6.3	8	44	45	39	99	217	1.1	3.9
289	51	1	1	0	1a	7.4	12	63	45	75	117	181	0.9	5.1
290	54	1	1	0	1b	16.3	12	102	45	108	78	88	1.2	4.1
291	59	1	1	0	1b	6.5	8	42	45	61	30	160	1.1	3.9

292	17	1	1	0	3a	5.5	6	38	45	65	36	246	1.0	3.9
293	55	1	1	0	1a	14.8	12	88	45	132	56	70	1.1	4.1
294	56	1	1	0	1a	5.3	13	39	45	56	24	204	1.1	3.5
295	57	1	1	0	1a	6.1	5	65	45	63	15	169	1.0	4.1
296	43	1	1	0	1b	48	12	125	45	85	64	34	1.5	3.7
297	59	1	1	0	1b	26.3	9	44	45	109	64	140	1.2	4.0
298	41	1	1	0	1a	6.3	18	49	45	86	81	236	1.0	4.9
299	54	1	1	0	3a	18.2	11	80	45	138	38	167	1.1	4.8
300	54	1	1	0	1a	12.2	15	70	45	86	296	169	1.0	3.3
301	51	1	1	0	3a	13.1	19	101	45	165	79	244	1.1	5.0
302	52	1	1	0	1b	11.8	14	21	45	10	37	213	0.9	5.6
303	63	1	1	0	1b	75	9	134	45	103	133	130	1.2	4.9
304	56	1	1	0	1a	56.1	12	123	45	83	229	126	1.3	5.1
305	51	1	1	0	1a	5.8	14	27	45	34	48	270	1.0	4.3
306	47	1	1	0	1b	3.6	18	24	45	22	27	243	1.0	3.9
307	54	1	1	0	3a	5	16	46	45	80	10	161	1.1	2.5
308	65	1	1	0	1a	11.8	14	161	45	181	872	220	1.1	4.6
309	61	1	1	0	1b	10.7	16	92	45	109	205	203	1.0	4.6
310	54	1	1	0	1b	7	18	48	45	111	48	208	1.1	4.8
311	48	1	1	0	3a	8.2	17	32	45	55	29	193	1.0	4.0
312	68	1	1	0	1a	5.2	16	38	45	27	70	204	0.9	4.2
313	51	1	1	0	1a	18.6	9	66	45	55	62	180	1.0	3.9
314	58	1	1	0	1a	9.6	17	43	45	29	10	286	1.0	4.3
315	56	1	1	0	1a	6.2	6	50	45	58	203	223	1.0	4.0
316	60	1	1	0	1b	8.4	19	122	45	168	66	166	1.1	4.7
317	62	1	1	0	1a	7.8	14	90	45	94	318	230	0.9	3.9
318	55	1	1	0	1a	6.7	18	119	45	292	310	184	1.0	4.9
319	37	1	1	0	1b	74.8	8	67	45	159	25	149	1.1	3.9
320	55	1	1	0	3a	6.1	16	22	45	33	17	192	0.9	4.6
321	51	1	1	0	3a	69.1	12	111	45	91	88	80	1.3	3.9
322	62	1	1	0	4	23.1	19	40	45	56	64	109	1.1	4.4
323	63	1	1	0	1b	23.1	16	152	45	118	235	83	1.1	4.5
324	62	1	1	0	2b	5.9	9	23	45	22	26	120	1.0	4.9

325	43	1	1	0	1a	4.2	12	44	45	80	180	246	0.9	4.2
326	52	1	1	0	1a	6.8	10	48	45	76	62	251	0.9	4.7
327	42	1	1	0	1a	27	19	32	45	38	148	246	1.0	4.1
328	53	1	1	0	3a	11.8	11	43	45	94	96	115	1.0	4.6
329	62	1	1	0	1a	4.1	5	38	45	38	102	211	1.0	4.0
330	49	1	1	0	3a	43.5	8	135	45	89	71	61	1.2	4.6
331	41	1	1	0	3a	14.3	9	37	45	72	92	127	0.9	3.9
332	45	1	1	0	1b	39.7	11	72	45	62	234	82	1.1	3.8
333	51	1	1	0	3a	16.5	10	150	45	212	59	67	1.1	4.2
334	44	1	1	0	3a	7.2	15	266	45	263	66	196	0.9	3.9
335	61	1	1	0	2	4.6	9	28	45	14	20	278	1.2	5.1
336	62	1	1	0	1a	4.4	8	22	45	31	24	276	0.9	4.9
337	57	1	1	0	4	43.5	6	96	45	160	367	151	1.1	3.8
338	55	1	1	0	1a	75	9	86	45	42	224	61	1.6	3.7
339	56	1	1	0	1a	4.8	8	42	45	62	25	169	1.2	4.6
340	55	1	1	0	1a	6.3	6	43	40	57	107	243	X	4.8
341	30	1	1	0	1a	5.6	11	66	40	119	138	387	0.9	X
342	22	1	1	0	3a	3.5	9	33	40	33	39	154	1.0	X
343	30	1	1	0	3a	4.4	14	26	40	25	17	228	1.0	X
344	25	1	1	0	1b	5.4	15	37	40	62	51	307	1.0	4.5
345	23	1	1	0	1a	3.8	11	108	40	352	48	198	1.0	X
346	31	1	1	0	1b	5.9	12	165	40	69	34	257	1.0	4.7
347	31	1	1	0	1a	8.6	13	25	40	37	12	230	1.1	3.9
348	25	1	1	0	3a	6.6	14	57	36	155	60	194	1.1	3.8
349	43	1	1	0	3a	4.6	7	78	36	152	25	229	X	3.2
350	27	1	1	0	3a	6.9	10	104	45	210	101	186	1.1	2.7
351	44	1	1	0	1a	5.4	6	36	45	56	61	250	1.0	4.1
352	47	1	1	0	1a	5.7	11	92	40	138	80	224	1.0	4.9
353	46	1	1	0	1a	4	8	36	40	73	39	219	1.0	5.8
354	41	1	1	0	1a	4.9	16	22	40	43	46	267	0.9	5.1
355	31	1	1	0	3a	4.2	7	149	45	309	71	190	1.1	4.1
356	41	1	1	0	1a	6.1	13	15	40	28	21	230	1.0	3.9
357	33	1	1	0	1a	4.1	12	23	40	51	29	199	1.0	X

358	66	1	1	0	1a	11.6	7	38	36	32	34	151	1.1	3.3
359	24	1	1	0	3a	4.3	12	36	40	135	113	192	1.1	3.9
360	28	1	1	0	3a	4.9	6	94	40	172	16	230	1.1	4.4
361	28	1	1	0	3a	3.5	11	129	40	72	34	213	1.0	5.6
362	34	1	1	0	1a	5.2	15	42	36	64	32	234	1.1	5.5
363	34	1	1	0	1a	7.3	11	97	36	125	69	196	1.1	4.1
364	52	1	1	0	2a/2c	3.7	8	23	40	32	51	222	1.0	5.2
365	32	1	1	0	1a	4	3	71	45	123	123	240	1.0	5.0
366	51	1	1	0	1a	5.5	7	25	40	36	64	248	X	X
367	23	1	1	0	1a	6.8	12	54	40	149	75	306	1.0	X
368	47	1	1	0	2b	4.3	9	45	45	62	30	254	X	X
369	59	1	1	0	6	3.7	8	32	40	36	20	169	1.1	X
370	48	1	1	0	3a	14.5	3	325	45	310	208	200	1.4	X
371	41	1	1	0	2a/2c	4.4	11	30	40	41	22	200	1.0	5.0
372	24	1	1	0	3a	7.6	5	33	45	25	22	236	1.1	4.6
373	45	1	1	0	1a	7.8	10	80	45	121	150	261	1.1	5.1
374	56	1	1	0	1a	7.6	7	86	40	125	48	165	1.1	3.6
375	34	1	1	0	1a	4.9	6	31	36	66	42	258	1.1	5.9
376	42	1	1	0	1a	3.7	14	50	45	39	58	231	1.0	5.5
377	34	1	1	0	1a	7.4	11	72	36	138	51	335	1.1	7.9
378	31	1	1	0	1a	3.6	8	38	40	51	38	266	1.0	X
379	44	1	1	0	3a	6.1	8	52	36	51	46	209	1.0	X
380	34	1	1	0	1a	6.3	11	54	45	68	44	224	1.1	X
381	28	1	1	0	3a	5.1	8	25	40	37	50	187	1.0	5.8
382	35	1	1	0	2	6	12	51	40	108	63	176	1.1	5.3
383	37	1	1	0	1a	4	13	65	36	79	61	250	1.0	4.8
384	38	1	1	0	3a	7.7	8	80	36	177	55	154	X	X
385	43	1	1	0	1a	6.7	10	41	40	45	34	261	1.0	4.6
386	35	1	1	0	3a	12	21	99	40	246	98	212	1.1	X
387	56	1	1	0	1a	4.9	4	35	40	59	30	146	1.1	4.0
388	33	1	1	0	1a	5.6	4	73	40	158	45	253	X	5.1
389	39	1	1	0	3a	14.3	6	207	40	395	238	127	1.2	3.9
390	35	1	1	0	3a	6.2	5	37	40	54	51	180	1.0	4.3

391	42	1	1	0	3a	7	10	53	40	62	51	185	1.1	2.6
392	26	1	1	0	1a	6.1	7	113	40	264	214	229	1.1	4.3
393	31	1	1	0	3a	4.4	11	63	40	157	47	293	1.1	3.0
394	36	1	1	0	3a & 1a	7.1	1	29	40	51	21	286	1.0	3.1
395	29	1	1	0	1a	4.5	11	143	40	222	318	234	1.1	3.6
396	43	1	1	0	1a	7.1	10	53	40	89	74	209	1.0	4.4
397	31	1	1	0	1a	6.1	10	46	40	62	112	236	X	3.8
398	35	1	1	0	3a	6.1	11	48	40	80	28	201	1.0	3.9
399	38	1	1	0	1a	4.4	11	76	40	167	49	209	1.0	4.2
400	34	1	1	0	1a	6.7	9	49	40	74	57	218	1.0	4.0
401	36	1	1	0	3a	3.5	20	68	40	96	60	235	1.0	2.3
402	29	1	1	0	1a	4.7	11	22	40	31	21	199	1.1	4.3
403	40	1	1	0	3a	6.5	15	60	40	111	37	161	1.0	4.6
404	33	1	1	0	3a	6.3	8	39	40	37	88	249	1.1	4.3
405	33	1	1	0	3a	5.3	13	42	40	69	40	234	1.0	2.5
406	53	1	1	0	1b	75	2	69	45	44	151	75	1.3	3.3
407	60	1	1	0	3a	20.3	13	97	45	103	56	53	1.4	4.0
408	47	1	1	0	1a	6.9	7	49	40	68	32	295	X	X
409	64	1	1	0	1a	46.4	12	105	45	151	124	224	1.0	3.3
410	52	1	0	1	3a	66.4	4	204	45	156	226	128	1.1	X
411	48	1	0	1	3a	11.7	6	79	45	117	79	193	1.1	X
412	50	1	1	0	1a	4.3	14	27	45	22	46	194	1.1	4.1
413	58	1	1	0	3a	12.4	6	50	36	59	34	163	1.1	2.1
414	38	1	1	0	3a	8.8	1	41	40	48	50	283	1.1	2.2
415	63	1	1	0	1b	22.8	14	78	36	68	107	90	1.2	5.6
416	54	1	1	0	1a	4	18	28	36	34	27	240	1.0	5.7
417	34	1	1	0	3a	20.9	3	162	45	258	241	136	1.2	4.3
418	38	1	1	0	3a	10.4	6	197	36	332	93	160	1.1	X
419	29	1	1	0	1a	4.6	9	43	36	90	19	226	1.1	4.3
420	25	1	1	0	3a	3.6	11	25	36	25	29	235	1.0	5.5
421	34	1	1	0	unknown	5.3	13	32	45	35	45	266	1.1	3.3
422	45	1	1	0	1a	4.9	8	30	40	56	131	278	1.0	4.9
423	36	1	1	0	3a	5.3	8	60	36	58	53	151	1.1	5.2

424	48	1	1	0	3a	6.7	9	71	40	58	52	107	1.1	X
425	45	1	1	0	3a & 1a	25.1	4	74	40	177	198	258	1.1	2.0
426	34	1	1	0	3a	3.7	11	66	40	164	179	365	1.0	X
427	30	1	1	0	1a	6.4	14	35	45	77	38	270	1.0	5.3
428	22	1	1	0	3a	7.8	10	42	45	66	31	171	1.1	X
429	40	1	1	0	1a	3	13	41	45	36	95	181	1.0	3.7
430	36	1	1	0	3a	75	10	36	40	92	31	139	1.0	3.7
431	37	1	1	0	1a	4.3	9	54	40	95	19	147	1.1	5.7
432	47	1	1	0	1a	27.4	3	132	45	101	105	107	1.3	X
433	46	1	1	0	1a	7.2	8	49	36	83	104	177	1.0	4.8
434	56	1	0	2	2b	16	13	118	40	140	87	82	1.2	2.6
435	32	1	1	0	3a	4.5	11	32	40	61	78	310	1.1	3.5
436	58	1	1	0	3a	26	8	167	40	209	76	199	1.2	2.2
437	25	1	1	0	1a	5.4	13	75	40	113	29	212	1.1	2.8
438	30	1	1	0	1a/1b	4.5	18	54	40	90	22	195	1.0	3.8
439	51	1	1	0	1a	4	15	45	36	54	16	230	1.2	4.9
440	40	1	1	0	1a	6.7	6	41	36	70	40	189	1.1	4.6
441	47	1	1	0	1a	4.4	9	44	45	26	302	268	1.0	X
442	48	1	1	0	3a	16.9	8	100	40	161	185	145	1.2	2.2
443	38	1	0	1	1a	10.2	5	68	40	94	80	188	1.0	6.0
444	43	1	1	0	1a	4.9	10	34	45	28	12	212	1.1	X
445	43	1	1	0	3a	5.5	5	25	40	41	52	296	1.1	4.7
446	34	1	1	0	3a	5.3	8	28	40	53	28	160	1.0	4.2
447	27	1	1	0	3a	5.8	12	45	40	41	3	258	1.1	4.1
448	35	1	1	0	1b	5.1	10	21	40	35	22	240	1.1	3.7
449	32	1	1	0	3a	10.6	8	69	40	119	40	175	X	4.4
450	58	1	1	0	3a	5.2	6	78	36	143	22	226	1.1	X
451	50	1	1	0	1a	11.8	15	113	40	57	55	232	1.0	4.4
452	32	1	1	0	1a	7.1	7	85	40	135	137	232	1.1	X
453	45	1	1	0	1a	6.6	6	26	40	47	47	220	1.0	5.5
454	31	1	1	0	1 & 3	8.6	6	26	40	42	48	329	1.0	5.8
455	45	1	1	0	3a	9.8	16	140	40	229	48	155	1.0	3.3
456	43	1	1	0	1a	40.3	8	55	40	96	249	100	1.1	4.1

457	49	1	1	0	1a	67.8	4	153	40	199	301	147	1.1	4.0
458	46	1	1	0	1b	10.4	3	37	45	38	125	129	X	2.9
459	28	1	1	0	1a	3.7	3	18	40	23	20	180	1.2	X
460	45	1	1	0	1a	10	9	73	40	96	38	271	1.0	5.4
461	33	1	1	0	1a	6.1	7	38	36	47	33	166	1.0	4.0
462	22	1	1	0	1b	7.3	12	81	36	234	41	189	1.1	3.2
463	41	1	1	0	1a	6.1	2	71	45	140	61	186	1.2	2.8
464	28	1	1	0	1a	7.6	9	73	40	179	39	156	1.0	5.2
465	45	1	1	0	3a	17	8	44	40	57	79	148	1.1	3.8
466	53	1	1	0	1a	6	7	27	40	50	24	232	1.1	6.3
467	59	1	1	0	3a	14.3	10	87	36	125	36	75	1.4	4.0
468	46	1	1	0	3a	14.3	4	72	40	104	59	206	1.1	4.1
469	49	1	1	0	3a	8.8	3	56	36	109	106	301	1.2	4.9
470	24	1	1	0	1a	6	13	45	45	95	31	247	1.1	4.9
471	52	1	1	0	1a	48	3	41	40	42	40	78	1.4	2.5
472	45	1	1	0	3a	11.9	2	88	40	140	51	142	1.1	3.9
473	41	1	1	0	3a	8.9	7	60	35	106	33	276	1.0	5.0
474	38	1	1	0	3a	9.4	16	41	36	45	26	219	1.0	4.1
475	41	1	1	0	3a	7.6	4	42	45	69	27	181	1.2	2.6
476	31	1	1	0	3a	3.7	8	52	35	62	61	306	1.0	5.7
477	25	1	1	0	1a	4	20	57	45	240	41	186	1.1	4.7
478	46	1	1	0	3a	6.9	4	25	36	41	44	164	1.0	4.8
479	23	1	1	0	3a	6.8	13	87	45	172	29	287	1.2	3.2
480	28	1	1	0	1a	5.8	7	199	45	67	21	213	1.1	5.0
481	54	1	1	0	1a	32.6	1.7 iqr	167	40	114	74	31	1.4	X
482	32	1	1	0	1a	5.8	14	22	40	59	83	253	1.0	5.3
483	54	1	1	0	3a	5.1	10	50	36	68	26	278	1.0	5.6
484	22	1	1	0	1a	4.3	5	176	45	241	106	370	1.2	5.1
485	40	1	1	0	1a	4.8	13	21	40	30	34	264	1.0	3.9
486	46	1	1	0	1a	48.8	3	88	40	108	121	115	1.1	3.8
487	41	1	1	0	3a	15.4	18	81	30	109	156	222	1.1	3.2
488	50	1	1	0	1b	5	8	51	40	94	193	357	0.9	5.0
489	40	1	1	0	3a	15.4	4	167	45	199	85	205	1.1	2.6

490	22	1	1	0	3a	5.3	8	82	45	305	35	165	1.2	3.4
491	25	1	1	0	1a	8.4	12	34	40	57	33	226	1.1	3.5
492	53	1	1	0	3a	4.4	11	25	40	38	53	235	1.1	X
493	25	1	1	0	1a	6	5	43	40	41	32	287	1.1	X
494	38	1	1	0	3a	5.3	8	109	40	243	148	265	1.1	X
495	36	1	1	0	1a	6.7	13	55	40	104	261	331	0.9	6.0
496	26	1	1	0	1a	5.3	6	129	40	351	44	222	1.2	3.3
497	40	1	1	0	1a	16.8	4	101	45	167	111	233	1.2	3.2
498	34	1	1	0	3a	6.1	9	40	40	75	36	256	1.0	X
499	28	1	1	0	1a	13.4	14	70	40	112	129	194	0.9	X
500	33	1	1	0	3a	8.2	6	217	45	385	38	306	X	5.6
501	61	1	1	0	1a	8.8	3	37	40	51	36	207	X	5.4
502	28	1	1	0	3a	5.6	5	180	40	573	74	255	1.1	X
503	23	1	1	0	1a	7.7	4	69	40	199	33	179	1.1	3.9
504	40	1	1	0	3a	5.5	7	19	40	35	41	186	1.1	X
505	37	1	1	0	3a	8.6	8	90	40	198	51	219	0.9	2.4
506	25	1	1	0	3a	10.4	6	167	40	605	110	167	1.1	X
507	32	1	1	0	1a	6.1	13	63	40	155	77	223	1.0	4.3
508	33	1	1	0	3a	5.9	15	60	40	136	49	175	1.0	4.7
509	33	1	1	0	3a	5.9	15	60	40	136	49	175	1.0	4.7
510	26	1	1	0	1a	4.9	18	48	36	103	43	246	0.9	4.3
511	33	1	1	0	1a	6.9	9	50	45	53	44	223	1.1	5.0
512	45	1	1	0	1a	35.3	3	62	45	68	189	146	1.2	3.4
513	37	1	1	0	1a	6.9	12	52	45	86	81	190	1.1	5.2
514	36	1	1	0	1a	7.1	10	21	45	15	43	254	1.0	5.0
515	40	1	1	0	3a	22	17	105	40	150	97	219	1.1	4.0
516	46	1	1	0	1b	6.1	11	55	40	89	32	186	0.9	3.8
517	32	1	1	0	3a	4.9	14	25	40	55	21	258	1.0	4.5
518	35	1	1	0	1a	6.7	9	201	40	106	144	256	0.9	7.4
519	41	1	1	0	3a	6.3	6	41	40	94	91	265	1.0	4.5
520	44	1	1	0	1a	5.3	11	85	36	163	65	263	1.0	5.4
521	35	1	1	0	3a	3.4	6	33	40	48	46	274	1.0	4.3
522	48	1	1	0	1a	7.2	11	56	36	62	192	269	1.0	4.1

523	41	1	1	0	1a	6.9	10	45	40	71	263	218	1.0	4.7
524	30	1	1	0	3a	5	16	42	40	78	50	303	1.0	5.4
525	35	1	1	0	2b	5.5	7	25	45	21	13	268	1.0	3.4
526	31	1	1	0	3a	4.3	14	50	36	64	134	176	1.0	4.5
527	23	1	1	0	3a	4.8	4	150	45	160	30	202	1.2	4.9
528	34	1	1	0	3a	3.7	7	52	40	85	86	301	1.0	X
529	48	1	1	0	1a	5.3	9	35	40	43	13	162	1.1	X
530	43	1	1	0	3a	7.7	12	44	40	88	31	362	1.0	4.7
531	22	1	1	0	3a	3.5	9	33	40	33	39	154	1.0	X
532	25	1	1	0	1a	6.8	9	42	40	50	32	235	1.1	3.6
533	46	1	0	1	1a	5.4	13	39	40	45	52	218	1.0	X
534	44	1	1	0	3a	10.2	8	104	45	140	93	200	1.1	4.0
535	45	1	1	0	1a	11.3	17	67	40	106	41	170	1.1	4.3
536	30	1	1	0	1b	7.6	5	68	40	77	153	268	0.9	X
537	42	1	1	0	3a	8.6	10	43	36	71	128	304	0.9	4.9
538	51	1	1	0	3a	5.3	0.7 iqr	80	36	154	39	218	1.2	4.8
539	43	1	1	0	1a	6.4	8	34	45	34	64	250	1.1	5.0
540	53	1	1	0	1b	8.9	19	48	40	78	259	269	1.0	X
541	24	1	1	0	3a	6.9	4	56	40	155	77	203	1.0	X
542	44	1	1	0	1a	14.6	8	53	40	57	114	206	1.2	X
543	46	1	1	0	1b	6.4	11	81	40	190	68	284	1.0	X
544	41	1	1	0	3a	14.5	8	78	45	67	208	212	1.1	3.9
545	30	1	1	0	3a	8.9	5	67	40	104	48	249	1.0	4.8
546	27	1	1	0	1a	6.3	5	78	40	135	59	220	1.0	X
547	43	1	1	0	1a	10.1	4	24	45	20	17	248	1.3	3.9
548	26	1	0	1	3a	27	2.6	98	45	148	38	152	1.2	X
549	33	1	1	0	1a	4.8	6	41	40	104	82	196	1.0	X
550	46	1	1	0	1a	25.7	9	81	40	141	71	145	1.1	X
551	55	1	1	0	2b	6.3	11	33	45	36	18	196	1.2	4.3
552	36	1	1	0	1a	11.8	5	66	45	119	48	133	1.2	4.2
553	49	1	1	0	1a	4.7	6	32	40	58	33	225	0.9	X
554	31	1	1	0	3a	5	8	44	40	131	45	270	1.0	X
555	42	1	1	0	3a	17.3	0	232	40	301	119	226	1.1	X

556	46	1	1	0	3a	5.9	9	29	40	54	96	288	1.0	4.0
557	35	1	1	0	1a	4.4	11	53	40	53	52	195	1.0	3.0
558	48	1	1	0	3a	10.3	6	108	40	197	50	163	1.0	2.3
559	42	1	1	0	1a	3.8	3	93	40	234	34	219	1.0	X
560	33	1	1	0	3a	8.8	5	57	40	92	686	217	1.0	5.3
561	35	1	1	0	1a	4.4	7	38	45	56	36	263	1.1	5.5
562	30	1	1	0	1a	6.6	2	70	40	138	49	190	1.1	X
563	23	1	1	0	3a	5.5	9	123	40	220	39	232	1.0	X
564	24	1	1	0	3a	8.1	10	76	40	113	71	215	1.1	X
565	44	1	1	0	1a	13.9	3	145	40	164	140	147	1.0	4.2
566	22	1	1	0	1a	6.1	3	108	40	228	101	298	1.0	X
567	28	1	1	0	3a	5.9	5	59	40	109	49	237	1.0	3.0
568	40	1	1	0	3a	4	10	46	40	89	157	267	1.0	4.3
569	39	1	1	0	3a	8.3	11	69	40	109	60	173	0.9	X
570	43	1	1	0	1a	4.7	11	53	40	71	28	212	1.0	X
571	36	1	1	0	3a	6.2	13	104	36	176	14	169	1.2	3.2
572	47	1	1	0	1a	4.4	5	63	40	123	242	196	1.0	3.5
573	29	1	1	0	3a	7.3	5	106	40	304	63	286	X	4.6
574	38	1	1	0	1a	11.2	12	150	40	223	48	228	1.1	X
575	33	1	1	0	1a	5	10	68	40	161	29	202	1.1	4.5
576	55	1	1	0	1b	5.4	8.8	50	40	58	17	154	1.1	6.8
577	25	1	1	0	1b	4.7	11	35	40	61	26	289	1.1	X
578	49	1	1	0	3a	6.8	4	34	45	39	19	278	1.2	3.3
579	48	1	1	0	4	4.7	6	38	40	86	77	236	1.0	6.1
580	22	1	1	0	1a	6.6	11	74	40	209	54	261	1.0	3.9
581	48	1	1	0	3a	10.4	4	48	40	140	160	154	1.0	3.9
582	40	1	1	0	3a	8	11	36	40	71	43	232	1.1	5.8
583	38	1	1	0	1a	3.7	8	20	40	27	15	182	1.0	4.7
584	30	1	1	0	1a	6	12	88	40	151	25	192	1.0	X
585	25	1	1	0	3a	5.4	13	62	36	77	30	172	1.1	X
586	37	1	1	0	3a	5.4	7	47	40	80	37	178	1.0	X
587	23	1	1	0	1a	4.6	13	30	40	43	42	200	1.0	X
588	47	1	1	0	1a	4	8	18	40	25	47	232	1.0	4.8

589	42	1	1	0	1a	7.8	10	56	40	92	30	227	1.1	X
590	52	1	1	0	3a	10.1	5	79	40	87	80	160	1.1	X
591	23	1	1	0	2b	4.9	8	56	40	126	59	316	1.0	3.9
592	33	1	1	0	1a	5	10	33	40	65	324	175	1.0	5.9
593	34	1	1	0	3a	6	7	38	45	69	44	212	X	4.3
594	24	1	1	0	1a	4.5	9	27	40	35	41	200	1.0	4.0
595	40	1	1	0	1a	10.5	10	126	40	242	307	149	1.2	X
596	38	1	1	0	3a	7.8	10	30	40	58	148	251	X	4.2
597	54	1	1	0	1a	21.7	8	101	40	97	245	176	1.0	2.9
598	35	1	1	0	1a	6.8	7	32	45	37	79	280	0.9	5.2
599	31	1	1	0	3a	6.8	7	35	40	49	51	247	1.0	X
600	31	1	1	0	1a	5.5	5	52	40	131	47	312	1.0	X
601	29	1	1	0	1a	7.4	8	409	45	231	23	212	1.0	4.1
602	23	1	1	0	1a	3.8	11	26	40	40	25	235	1.1	4.1
603	33	1	0	1	1a	12	9	73	40	144	120	139	1.0	X
604	44	1	1	0	3a	3.3	9	51	40	88	42	184	1.0	X
605	34	1	1	0	1a	6.1	10	89	40	185	115	188	1.0	X
606	53	1	1	0	3a	7.3	8	110	40	160	107	208	1.1	3.9
607	49	1	1	0	3a	14	11	102	45	155	79	138	1.1	5.4
608	61	1	1	0	3a	10.1	5	57	40	112	52	264	1.2	3.2
609	45	1	1	0	1a	5.3	17	34	40	61	75	194	1.0	X
610	27	1	1	0	3a	8.8	3	101	40	201	61	256	1.0	3.0
611	38	1	1	0	3a	6	3	20	36	20	25	317	1.1	4.3
612	41	1	1	0	3a	26.3	11	133	40	240	153	123	1.2	2.8
613	26	1	1	0	3a	8	11	20	40	20	19	359	1.1	4.5
614	37	1	1	0	1a	4.6	9	34	40	62	74	182	1.1	X
615	49	1	1	0	1a	46.6	3	79	36	50	96	68	1.5	3.3
616	50	1	1	0	1b	4.6	7	43	45	51	28	256	1.1	5.3
617	38	1	1	0	3a	7.5	5	26	40	31	43	174	1.0	X
618	44	1	1	0	1a	5.6	8	83	45	143	32	211	1.1	X
619	34	1	1	0	1a	8.8	9	570	40	1368	115	264	1.0	4.1
620	30	1	1	0	1a	10.5	8	54	45	92	113	253	1.0	4.9
621	48	1	1	0	3a	7.5	11	64	45	97	109	171	1.2	5.0

622	31	1	1	0	3a	5.6	14	40	40	71	55	213	1.0	X
623	42	1	1	0	3a	3.9	8	46	40	105	62	256	1.0	6.5
624	46	1	1	0	3a	13.4	4	143	40	316	1301	130	1.0	X
625	46	1	1	0	3a	14.3	13	137	40	236	163	175	1.2	X
626	28	1	1	0	1a	7	19	73	40	165	48	261	1.1	3.4
627	34	1	1	0	3a	8.8	11	40	40	106	39	257	1.1	2.5
628	55	1	1	0	1a	6.7	16	51	40	89	59	224	1.4	3.2
629	29	1	1	0	3a	8.6	3	25	40	24	18	297	1.1	X
630	39	1	1	0	1a	3.7	16	26	40	44	35	247	1.1	5.0
631	45	1	1	0	1a	5.3	6	54	40	87	70	256	1.0	X
632	43	1	1	0	1a	5.4	7	41	40	83	28	242	1.0	X
633	41	1	1	0	1a	7.6	12	81	45	137	165	172	1.1	4.0
634	31	1	1	0	3a	5.2	6	72	40	94	24	253	1.0	X
635	31	1	1	0	3a	5.3	6	50	40	100	29	232	1.1	5.2
636	30	1	1	0	3a	8.3	13	43	30	60	97	242	1.0	3.3
637	36	1	1	0	3a	4.3	7	57	40	137	39	214	1.0	4.4
638	25	1	1	0	1a	5.3	8	54	40	86	220	264	1.0	3.6
639	47	1	1	0	1a	6.9	7	49	40	68	32	295	X	X
640	26	1	1	0	3a	8.8	3	39	40	79	52	321	1.0	X
641	39	1	1	0	1a	5.6	4	27	45	22	60	251	X	8.9
642	49	1	1	0	1a	7.9	14	35	40	55	40	188	1.0	X
643	47	1	1	0	1a	10.3	4	160	40	282	259	186	1.0	3.4
644	34	1	1	0	1a	6.5	15	56	40	80	81	276	1.0	4.3
645	43	1	1	0	3a	7.7	9	159	40	331	102	226	1.1	X
646	29	1	1	0	1b & 3a	6.1	11	99	40	280	57	171	1.1	X
647	28	1	1	0	2b	8.8	5	25	40	19	16	280	1.1	3.6
648	54	1	1	0	3a	11.7	4	133	40	183	589	196	1.1	5.2
649	34	1	1	0	1a	6.8	1	102	40	315	357	232	1.0	X
650	25	1	1	0	1a	6.2	6	61	40	95	41	234	1.0	X
651	34	1	1	0	1a	5.8	9	32	36	25	14	219	1.0	4.7
652	51	1	1	0	1b	9.1	14	41	45	29	16	135	1.2	5.5
653	37	1	1	0	3a	2.9	14	53	45	36	56	207	1.0	X

654	58	1	1	0	1b	6.9	12	44	40	71	34	235	X warfari n	4.9
655	33	1	1	0	3a	13.7	6	72	40	178	66	168	1.0	1.0
656	35	1	1	0	3a	9.3	9	56	40	99	112	313	1.1	4.2
657	41	1	1	0	1a	4.8	8	52	40	75	70	220	1.0	5.3
658	37	1	1	0	1a	6.6	6	61	45	32	14	302	1.1	4.8
659	34	1	1	0	1a	4.8	19	25	40	62	49	383	1.0	X
660	35	1	1	0	6	5.4	5	34	40	94	91	169	0.9	4.7
661	23	1	1	0	1a	6.9	8	94	45	162	81	208	1.1	X
662	38	1	1	0	3a	3.5	14	42	40	68	64	344	1.0	6.4
663	46	1	1	0	3a	3.4	15	38	40	46	52	244	0.9	5.1
664	41	1	1	0	1a	5.8	12	79	40	104	308	257	1.1	X
665	42	1	1	0	3a	4.5	9	24	40	34	73	284	1.0	3.8
666	28	1	1	0	1b	6.6	11	83	40	203	53	228	1.0	2.7
667	46	1	1	0	4	40.9	9	158	45	128	115	120	1.2	2.9
668	37	1	1	0	3a	6	5	49	45	64	61	208	1.1	3.9
669	46	1	1	0	1a	6	7	61	40	76	38	189	1.1	2.9
670	31	1	1	0	1a	6.7	6	53	36	96	81	269	0.9	4.4
671	44	1	1	0	1a	17.3	2	140	45	157	167	162	1.0	3.2
672	23	1	1	0	1a	7.6	12	38	40	56	17	236	1.1	X
673	48	1	1	0	1a	6.2	10	20	45	34	26	243	1.1	4.7
674	48	1	1	0	3a	9.2	4	155	40	87	88	128	1.2	X
675	32	1	1	0	3a	4.6	12	24	40	39	24	148	1.0	3.7
676	28	1	1	0	3a	7.8	8	66	40	145	56	156	1.0	2.9
677	34	1	1	0	3a	7.9	8	45	40	87	62	241	1.0	X
678	49	1	1	0	3a	7.6	7	52	45	40	76	383	1.1	4.1
679	26	1	1	0	1a	4.9	6	74	36	136	132	260	1.0	3.7
680	40	1	1	0	3a	11.8	3	83	40	134	43	222	1.0	2.8
681	35	1	1	0	3a	5.8	7	110	40	253	36	175	1.1	3.1
682	23	1	1	0	1a	6	7	133	40	227	53	187	1.0	4.6
683	23	1	1	0	3a	6.1	13	31	40	41	17	165	1.0	X
684	34	1	1	0	3a	3.7	7	52	40	85	86	301	1.0	X
685	21	1	1	0	unknown	4.8	6	22	40	43	40	310	1.0	X

686	33	1	1	0	1a	5	13	136	40	267	50	262	1.1	3.5
687	35	1	1	0	unknown	4.6	11	27	36	29	37	265	1.0	6.9
688	49	1	1	0	3a	5.9	8	39	40	68	37	269	1.0	3.6
689	33	1	1	0	3a	4.3	9	95	36	92	16	210	X	3.9
690	35	1	1	0	1b	5	10	36	40	66	95	371	1.0	X
691	47	1	1	0	3a	15.9	11	143	40	247	76	195	1.2	2.1
692	51	1	1	0	3a	46.4	10	151	40	153	260	168	1.2	X
693	54	1	1	0	1a	6.2	6	18	40	21	45	217	1.0	6.1
694	37	1	1	0	3a	7.7	10	37	40	66	44	383	0.9	3.0
695	34	1	1	0	1a	6.8	4	36	45	49	374	175	1.1	5.4
696	36	1	1	0	2b	6	7	68	45	102	18	211	1.0	X
697	41	1	1	0	3a	4.4	11	80	45	147	31	161	1.0	X
698	36	1	1	0	1a	10.4	4	85	40	206	82	183	1.0	X
699	38	1	1	0	3a	6.3	17	61	40	99	20	194	X	4.8
700	44	1	1	0	3a	6.2	1	37	40	65	25	250	1.0	X
701	35	1	1	0	1a	7.9	9	22	40	55	76	240	1.0	X
702	37	1	1	0	1a	6.4	11	32	40	46	69	200	1.0	X
703	47	1	1	0	3a	26.6	12	61	45	93	426	203	1.0	4.8
704	31	1	1	0	3a	5.2	10	47	45	64	50	225	1.0	X
705	24	1	1	0	1a	11.8	7	61	40	164	42	292	1.2	X
706	49	1	1	0	1a	5.6	11	19	45	14	18	123	X	4.4
707	44	1	1	0	1a	7	4	39	40	122	155	152	1.0	3.9
708	28	1	1	0	3a	5.8	16	25	40	36	67	270	0.9	X
709	39	1	1	0	1a	4.7	11	29	45	29	63	248	1.0	X
710	45	1	0	2	1a	33.3	7	65	40	81	142	113	1.1	3.7
711	35	1	1	0	3a	5.6	9	55	40	84	18	304	1.2	2.6
712	28	1	1	0	1a	5.1	8	28	40	72	84	230	1.0	4.2
713	43	1	1	0	1a	16.6	5	27	40	37	91	190	1.1	5.1
714	36	1	1	0	1a	7.6	8	49	45	75	63	175	1.1	X
715	55	1	1	0	1b	11.2	5	94	40	203	86	289	1.0	3.7
716	28	1	1	0	1a	4.3	14	47	45	72	84	224	1.0	4.3
717	28	1	1	0	3a	10.4	2	33	40	104	74	171	1.0	X
718	29	1	1	0	1a	9.1	15	59	40	232	130	338	0.9	2.4

719	42	1	1	0	1a	3.8	2	19	40	37	18	269	1.1	X
720	60	1	1	0	2b	10.4	9	92	40	106	82	284	2.2	2.6
721	37	1	1	0	3a	8.5	10	76	40	114	108	174	0.9	X
722	31	1	1	0	1a	3.9	10	52	40	61	229	241	1.0	3.9
723	28	1	1	0	1a	5.1	4	38	40	52	65	292	1.0	X
724	30	1	1	0	1a	3.5	9	51	36	195	156	219	X	X
725	36	1	1	0	1	3.4	12	30	40	43	63	314	1.1	3.2
726	53	1	1	0	3a	6.7	0	54	40	57	48	285	1.1	3.5
727	57	1	1	0	2b	10	3	44	36	50	149	282	1.1	5.2
728	42	1	1	0	3a	6	8	37	40	29	273	244	0.9	X
729	28	1	1	0	1a	5.1	8	191	40	490	96	268	1.0	5.1
730	58	1	1	0	3a	25.1	7	171	45	148	91	220	1.1	X
731	25	1	1	0	1a	5.3	8	181	45	110	65	260	1.0	5.5
732	26	1	1	0	3a	5	8	31	40	51	30	302	1.0	3.4
733	53	1	1	0	3a	4	3	38	40	68	20	243	1.0	1.8
734	39	1	1	0	3a	5.9	8	35	40	66	97	224	1.1	4.8
735	27	1	1	0	3a	6.2	8	56	40	103	56	170	1.0	3.5
736	36	1	1	0	1a	8.3	11	30	40	43	82	319	1.0	5.6
737	42	1	1	0	1a	6.1	11	129	40	229	138	199	1.1	3.9
738	43	1	1	0	1a	7.8	4	69	40	80	64	190	1.1	4.0
739	30	1	1	0	3a	5.6	5	73	40	137	41	245	1.0	X
740	35	1	1	0	2	8.5	2	56	40	102	44	183	1.1	3.7
741	21	1	1	0	1a	9	9	51	40	94	183	284	1.0	2.8
742	42	1	0	1	unknown	8.5	9	27	40	45	20	280	1.0	4.6
743	22	1	1	0	1a	5.6	5	93	40	220	94	350	1.0	4.6
744	31	1	1	0	3a	7.2	10	33	40	76	57	347	0.9	X
745	20	1	1	0	1a & 3a	8.3	9	249	40	536	60	232	1.0	3.6
746	20	1	1	0	unknown	7.6	7	32	40	41	20	193	1.0	4.8
747	48	1	1	0	1b	6.6	12	70	40	63	80	238	1.0	X
748	22	1	1	0	unknown	4.3	2	51	40	96	33	299	X	3.5
749	21	1	1	0	3a	4.6	7	25	40	50	34	202	1.1	X
750	23	1	1	0	1a	8.2	12	48	40	85	75	219	1.0	5.3
751	41	1	1	0	1a	12.2	11	54	40	68	61	190	1.1	4.3

752	30	1	1	0	1a	9.1	9	66	40	87	18	275	1.1	2.9
753	21	1	1	0	unknown	6.1	2	30	40	44	29	172	1.1	3.6
754	31	1	1	0	3a	6.6	8	31	40	34	60	271	1.0	3.6
755	34	1	1	0	1a	5.1	6	64	40	79	35	220	1.0	4.0
756	25	1	1	0	3a	14.3	3	102	40	269	90	260	1.1	2.2
757	33	1	1	0	1a	5	13	267	40	136	50	262	1.1	3.5
758	37	1	1	0	unknown	5.4	8	25	40	47	20	254	1.0	X
759	37	1	1	0	1a	26.3	4	107	40	202	190	223	1.1	X
760	32	1	1	0	3a	7.8	12	122	40	359	72	272	1.0	X
761	35	1	1	0	1a	5.9	12	29	40	59	23	264	1.0	x
762	49	1	1	0	1a	4.2	11	33	40	87	30	200	1.1	X
763	25	1	1	0	1a	4.8	10	39	40	48	62	320	1.0	3.4
764	22	1	1	0	3a	7.4	6	102	40	86	27	207	1.0	3.5
765	26	1	1	0	3a	4.2	11	50	40	123	34	144	1.1	3.2
766	19	1	1	0	3a	9.9	8	23	40	30	51	249	1.1	3.9
767	20	1	1	0	1a	6	13	39	40	70	57	280	1.0	4.0
768	43	1	1	0	1b	21.3	1	65	40	78	129	106	1.2	X
769	28	1	1	0	3a	5.3	9	73	40	156	57	195	1.1	3.9
770	64	1	1	0	3a	30.3	17.5	89	40	64	33	90	1.1	X
771	38	1	1	0	1a	8.7	7	27	40	80	61	173	1.1	X
772	21	1	1	0	1a	7.4	9	42	36	95	26	267	1.0	3.9
773	25	1	1	0	3a	7.2	6	81	40	115	34	327	0.9	X
774	32	1	1	0	3a	7.6	7	50	40	61	67	329	1.1	X
775	33	1	1	0	1a	6.8	10	42	45	135	55	268	1.0	X
776	32	1	1	0	3a	5.8	8	195	40	518	400	169	1.1	5.5
777	43	1	1	0	1a	6.8	16	41	40	44	108	294	1.0	X
778	30	1	1	0	1a	4	10	48	40	249	102	280	1.1	X
779	49	1	1	0	3a	6.7	7	42	40	86	81	223	1.0	5.8
780	38	1	1	0	1a	6.6	9	64	45	94	82	256	1.1	4.1
781	48	1	1	0	3a	18.2	8	85	40	134	70	103	1.1	3.6
782	31	1	1	0	3a	3.6	4	23	40	41	17	408	1.1	X
783	21	1	1	0	1a	5.2	12	191	40	806	240	238	1.1	X
784	40	1	1	0	3a	6.4	9	42	40	65	36	264	1.1	3.2

785	29	1	1	0	3a	12	1	221	40	486	173	214	X	X
786	42	1	1	0	unknown	10.5	7	45	40	84	47	196	1.0	6.7
787	28	1	1	0	1b	5.9	7	29	40	45	20	334	1.1	X
788	48	1	1	0	3a	38	8	59	36	26	31	41	1.4	1.9
789	43	1	1	0	3a	5.9	14	65	36	123	38	192	1.1	4.5
790	51	1	1	0	3a	36.3	7	73	45	95	137	168	1.0	4.0
791	41	1	1	0	1a	11.6	5	61	40	100	84	273	1.0	5.2
792	26	1	1	0	3a	4.9	8	41	40	104	20	248	0.9	5.2
793	38	1	1	0	3a	13.9	5	186	36	211	127	219	1.2	3.9
794	63	1	1	0	1a	75	8	108	45	96	188	90	1.3	4.1
795	49	1	1	0	1a	29.9	10	143	36	187	84	110	1.1	X
796	35	1	1	0	1a	8	10	67	45	126	97	214	1.1	3.8
797	26	1	1	0	1b	6.1	8	21	40	23	63	271	1.0	5.2
798	32	1	1	0	1a	7	7	25	45	44	89	227	1.1	x
799	37	1	1	0	4	10	8	60	45	72	25	488	1.0	4.8
800	38	1	1	0	1a	14.1	13	41	45	87	77	179	1.0	5.8
801	45	1	1	0	3a	5.1	12	40	36	74	19	154	1.1	5.1
802	35	1	1	0	3a	4.7	13	77	45	141	50	222	1.0	X
803	24	1	1	0	3a	3.3	6	21	40	35	16	295	1.1	4.6
804	39	1	1	0	3a	9.2	5	53	40	102	20	190	1.1	3.1
805	45	1	1	0	1a	21.1	5	130	36	156	263	151	1.0	5.5
806	37	1	1	0	3a	5.4	11	45	40	82	34	271	1.0	3.8
807	42	1	1	0	3a	10	8	38	40	101	69	248	1.1	4.1
808	39	1	1	0	1a	7.6	5	127	36	331	158	165	1.0	4.1
809	27	1	1	0	3a	14.5	6	226	40	305	169	225	1.1	2.8
810	37	1	1	0	1a	11.6	5	58	36	85	121	145	1.0	4.4
811	54	1	1	0	1a	38.5	9	120	40	68	78	46	1.4	X
812	45	1	1	0	3a	46.4	14	182	40	344	129	102	1.1	3.8
813	51	1	1	0	3a	23.9	11	158	36	300	272	188	1.4	3.4
814	47	1	1	0	1a	11.8	19	104	45	216	103	217	1.0	3.9
815	32	1	1	0	3a	8.8	10	86	45	176	34	178	1.2	4.6
816	25	1	1	0	1a	6.3	13	205	45	438	146	344	1.0	3.4
817	26	1	1	0	1a	9	8	71	45	52	206	276	1.2	4.3

818	55	1	1	0	3a	8.4	11	85	36	148	59	272	1.2	3.7
819	38	1	1	0	1b	11.8	8	79	36	106	69	231	1.0	4.7
820	38	1	1	0	3a	11.9	8	211	36	393	83	378	1.2	4.9
821	38	1	1	0	3a	6.1	8	30	36	31	29	363	1.1	4.5
822	37	1	1	0	3a	13.8	7	83	40	78	79	172	1.2	5.2
823	33	1	1	0	1b	7.6	8	122	40	235	191	177	1.0	4.2
824	42	1	1	0	1a	8.5	8	48	45	71	62	119	X	5.3
825	40	1	1	0	1a	6.6	12	40	36	58	44	232	1.1	3.3
826	52	1	1	0	3a	14.5	20	75	45	42	75	42	1.5	X
827	54	1	1	0	1a	38.5	9	167	40	114	74	31	1.4	X
828	37	1	1	0	1a	11.6	5	58	36	85	121	241	1.0	4.4
829	53	1	1	0	1b	9.6	14	54	36	73	71	162	1.1	3.9
830	29	1	1	0	1a	7.4	12	120	36	315	49	280	1.1	3.2
831	35	1	1	0	1a	6.4	8	24	45	20	16	242	1.1	4.1
832	43	1	1	0	3a	6.1	11	73	36	109	42	159	1.0	3.7
833	34	1	1	0	1a	5.1	10	47	40	146	79	266	1.0	5.0
834	60	1	0	2	3a	9.5	13	93	45	126	44	156	1.0	4.4
835	64	1	1	0	3a	26.3	2	65	45	66	171	123	1.2	X
836	44	1	0	2	1b	6.8	3	71	45	70	85	169	1.1	X
837	59	1	1	0	3a	21.2	8	74	40	110	119	180	1.1	X
838	50	1	0	2	1a	5.9	10	26	45	25	18	273	1.1	4.9
839	60	1	1	0	3a	11.8	12	72	45	55	544	142	1.0	3.8
840	64	1	1	0	3a	8	16	48	45	46	22	148	1.0	4.4
841	48	1	1	0	3a	4.4	9	20	45	20	30	443	1.1	2.2
842	57	1	1	0	1a	33.3	8	58	45	22	99	109	1.5	2.8
843	33	1	1	0	3a	7.7	9	39	45	59	87	175	1.1	2.6
844	83	1	1	0	4	10.9	16	30	45	31	30	207	1.2	2.7
845	40	1	1	0	2b	4.3	12	20	45	18	26	247	1.2	4.1
846	55	1	0	2	1a	7.8	4	27	45	14	61	218	1.0	6.9
847	38	1	1	0	1a	8.9	21	30	45	72	70	203	1.1	X
848	58	1	1	0	3a	26.3	10	34	45	69	76	148	1.5	X
849	35	1	1	0	3a	5.3	15	59	45	111	70	348	1.0	3.5
850	31	1	1	0	1a	4.2	2	14	45	27	31	165	1.1	X
